# Mapping EQ-5D-5L Utilities in Dementia: Integrating Self and Proxy Reports

**DOI:** 10.1016/j.jval.2025.05.009

**Published:** 2025-08

**Authors:** Hannah Hussain, Anju Keetharuth, Allan Wailoo, Donna Rowen

**Affiliations:** 1 Office of Health Economics, London, England, UK; 2 Sheffield Centre for Health and Related Research, University of Sheffield, Sheffield, England, UK

**Keywords:** dementia, EQ-5D-5L, health-related quality of life, patient-reported outcomes, proxy-report, statistical mapping

## Abstract

**Objectives:**

EQ-5D is widely used in dementia research, but cognitive impairments often necessitate proxy assessments, resulting in differences between self- and proxy-reported data. Some EQ-5D dimensions are better reported by people with dementia (PwD), particularly in the mild to moderate stages, whereas others are more accurately captured by proxies. This study evaluates whether a combined utility value, integrating both PwD and proxy reports, can be predicted when data from only 1 respondent type are available.

**Methods:**

Data from 2 dementia studies, ACTIFCARE and EPIC, were used to develop mapping models aimed at predicting combined utility values. These models integrate dimension-specific EQ-5D-5L responses from both respondent types to enhance health-related quality-of-life (HRQoL) assessments. Response mapping with ordered probit models was used to predict EQ-5D-5L responses when only 1 respondent type’s data were available. Model performance was evaluated by comparing observed and predicted data across dementia severity stages.

**Results:**

Combined utility values provided a more accurate reflection of HRQoL, showing greater sensitivity to health status changes. Proxy data proved to be more effective in predicting PwD responses for certain EQ-5D-5L dimensions, suggesting that proxy data collection may be particularly useful in specific situations. The mapping models performed well for mild-to-moderate dementia but were less accurate in severe dementia because of limited data.

**Conclusions:**

Combined utility values improve HRQoL assessments, particularly in detecting subtle health changes in mild-to-moderate dementia. These models support their use in economic evaluations of dementia interventions, although challenges remain in severe dementia because of data limitations.

## Highlights

• This study addresses the evidence gap in dementia research by developing mapping models that integrate self- and proxy EQ-5D responses to predict combined utility values.
• Key findings show that combining self- and proxy-reported data improves health-related quality-of-life assessments, particularly in detecting subtle health status changes in mild-to-moderate dementia.
• These results have significant implications for healthcare decision making, enhancing the accuracy of economic evaluations for dementia interventions and informing better health outcomes assessments in health economics and outcome research practice.

## Introduction

Health-related quality-of-life (HRQoL) assessments are essential tools in clinical research and economic evaluation, providing insights into individual well-being and the impact of interventions. Selecting an appropriate HRQoL measure requires considering disease-specific and generic tools. Among these, EQ-5D is widely used in dementia research because of its conciseness, feasibility, and acceptability.^1^, ^2^, ^3^, ^4^

Dementia presents challenges for self-assessment of HRQoL because of cognitive impairments, often necessitating proxy assessments from caregivers or family members.^5^ Differences between self- and proxy-reports are well documented, with each source potentially providing valuable insights into the person with dementia’s (PwD) health.^6^, ^7^, ^8^ Relying on either one alone may be inappropriate. This study uses proxy reports from informal caregivers (eg, family members) in ACTIFCARE and professional caregivers (eg, care home staff) in EPIC, each offering unique insights into PwD health. Although proxies provide critical insights when cognitive impairments limit self-reporting, discrepancies such as proxies often reporting lower QoL scores have been noted.^9^ Who provides assessments impacts evaluations of dementia interventions and their ability to detect meaningful changes in QoL

HRQoL assessments must be accurate and sensitive enough to detect changes in condition, this information drives cost-effectiveness analyses that can affect policy and pricing decisions. Implementing a combined utility value could enhance the precision of HRQoL assessments by improving how accurately changes in health status are reflected within quality-adjusted life-year calculations.

Our previous research, published in Alzheimer’s and Dementia (March 2025), developed a combined utility based on EQ-5D-5L dimensions by respondent type (PwD or proxy). Empirical testing demonstrated that certain dimensions of HRQoL are more accurately reported by different respondent types. Specifically, PwD provided better assessments for “pain/discomfort” and “anxiety/depression,” whereas proxies more accurately reported “self-care” and “usual activities.” These findings form the basis for the combined utility approach of this study, as summarized in Appendix 1 in Supplemental Materials found at https://doi.org/10.1016/j.jval.2025.05.009.

Collecting HRQoL data from both PwD and proxies is challenging because of participant burden, lack of proxies, and cognitive impairments that limit PwD’s self-reporting abilities.^6^^,^^10^ This is particularly true in severe dementia, for which proxies commonly report quality-of-life outcomes, whereas self-reports are more typical in milder stage studies.^5^^,^^8^ It is well established that caregiver burden can influence proxy reporting, with greater burden often associated with more negative assessment of the PwD’s quality of life.^11^ Although our data set did not include caregiver burden score, this remains a key consideration for the interpretation and use of proxy data. The potential for caregiver burden to introduce systematic bias has important implications for methodological approaches that apply proxy data, including mapping algorithms.

This article applies statistical mapping to achieve a target combination of EQ-5D-5L dimensions (see Appendix Table 1 in Supplemental Materials found at https://doi.org/10.1016/j.jval.2025.05.009), when only 1 respondent type is available, enabling the use of combined utility values for economic evaluations of dementia interventions.

Mapping techniques link outcomes from different measures and are increasingly valuable when utility data are not directly collected.^12^ This is particularly useful in studies with disease-specific measures, aligning with the National Institute for Health and Care Excellence (NICE)’s preference for deriving utility estimates from EQ-5D for quality-adjusted life-year calculations.^13^ Two primary types of mapping exist: direct and indirect (or “response”) mapping. Direct mapping estimates the overall utility value from the source measure.^11^ Indirect mapping involves a 2-stage process: first, estimating response levels for each domain of the target measure, second, calculating the expected utility value by assigning probabilities to health states and their utilities.^14^

The aim of this study is to evaluate whether a predefined target-combined utility value can be accurately predicted using data from only 1 respondent type (either the PwD or proxy) through statistical mapping methods. Data from 2 existing dementia trials that collected EQ-5D-5L responses were used. The objective is to enable studies to generate target combination EQ-5D utility values by combining self- and proxy-reported EQ-5D-5L dimensions into a single health state and corresponding utility value, even when only 1 type of respondent data are available. This study examines the potential impact of using combined utilities in economic evaluations, a detailed explanation of the methodologies, and results for each EQ-5D-5L dimension, including utility values.

## Methods

### Study Data

Data from 2 nonpharmacological dementia studies, ACTIFCARE and EPIC, were used. Both studies collected EQ-5D-5L data from PwD and proxies but differed in their settings, methodologies, and data collection approaches:

• ACTIFCARE: a multinational observational study recruited community-dwelling PwD and informal caregivers as proxies. Data were collected through face-to-face interviews at baseline, 6 months, and 12 months. This study included measures such as the Clinical Dementia Rating (CDR), Mini Mental State Examination (MMSE), Neuropsychiatric Inventory, Personal and Social Performance Scale (PSMS), Instrumental Activities of Daily Living, and EQ-5D-5L.
• EPIC: A cluster-randomized controlled trial in care homes recruited institutionalized PwD and staff proxies. Data were collected through structured interviews and assessments at baseline, 6 months, and 16 months. Measures included the CDR, Neuropsychiatric Inventory-Nursing Home version, the Functional Assessment Staging Tool (FAST), and EQ-5D-5L.

Differences in data collection methods and settings may influence responses. Face-to-face interviews in ACTIFCARE likely capture personal perspectives, whereas structured assessments in DCM-EPIC provide professional insights. These contextual differences should be considered when evaluating dementia-related interventions.

The differences in residential settings, respondent types and dementia symptom measures necessitated distinct analyses and the target combination of dimension reports differed by residential status of the PwD (see Appendix Table 1 in Supplemental Materials found at https://doi.org/10.1016/j.jval.2025.05.009).

EQ-5D-5L is a widely used HRQoL measure, comprising 5 dimensions: mobility, self-care, usual activities, pain/discomfort, and anxiety/depression. Each dimension is rated across 5 levels of severity: level 1, no problems; level 2, slight problems; level 3, moderate problems; level 4, severe problems; and level 5, extreme problems/unable to perform. The development and preliminary testing of EQ-5D-5L demonstrated its utility in a variety of clinical and research settings.^15^

### Statistical Analysis

#### Impact of a combined utility value

Mean EQ-5D-5L utility values derived from PwD reports, proxy reports, and combined values based on target dimension reports are compared. This analysis was conducted using dyads with complete data, categorized by CDR stage, and paired samples student’s *t* tests were used to assess statistical significance. This step was conducted to examine the potential impact of applying a combined utility value.

#### Response mapping

We used response mapping, which generated predicted responses for each EQ-5D-5L dimension, for 3 reasons. First, there is no accepted EQ-5D-5L value set for the United Kingdom/England. To use direct mapping methods would require the EQ-5D-5L responses to be transformed to EQ-5D-3L values, using published mapping methods. The distribution of mapped values differs from directly observed data; therefore, the appropriate method for conducting subsequent second stage mappings is currently uncertain. Second, we wish to predict PwD EQ-5D-5L responses for some dimensions from proxy responses and vice versa for other dimensions. Response mapping reflects the data generating process for this requirement and enables researchers to use observed data for dimensions in which target respondent data are available and apply mapping methods only for dimensions in which predictions are needed. Third, response mapping provides estimates that can be used to apply all international EQ-5D-5L value sets.

Ordered probit models were estimated for each EQ-5D-5L dimension, predicting the likelihood of a respondent selecting each severity level based on PwD or proxy data. Cluster-robust standard errors adjusted for intrapatient correlations within PwD-proxy dyads, accounting for potential dependencies between reports.

#### Model development and evaluation

Dimension models included relevant variables and were refined based on magnitude, direction and significance of coefficients. Model fit was evaluated using the Akaike Information Criterion and Bayesian Information Criterion, in which lower values indicate better fit by balancing model complexity and goodness of fit.

Predictive margins focused on predicting EQ-5D-5L reports from one respondent type (either PwD or proxy) as a function of the other. Average Marginal Effects assessed the impact of each predictor variable on the probability of response levels within each EQ-5D-5L dimension. Average Marginal Effects provided insights into how changes in predictor variables, such as MMSE scores, affect the likelihood of different severity levels, ensuring that the model accurately captures the relationship between predictors and outcomes.

#### Comparison of observed and predicted frequencies

STATA’s “predict” command was used to generate predicted frequencies for each EQ-5D-5L dimension, compared with observed frequencies to evaluate model performance.

#### Utility value estimation

The second stage of the analysis involved assigning utility values to predicted EQ-5D-5L responses by mapping them onto the EQ-5D-3L value set, following NICE’s current recommendations for health technology assessments.^16^, ^17^, ^18^ Presented in the appendices are utility distributions based on value sets from other selected countries (Australia,^19^ United States,^20^ and The Netherlands^21^). This dual approach was used because the response mapping method allows 2-stage processes, enabling the application of multiple country-specific value sets, and was utilized to identify any potential interesting differences.

The combined utility score was derived by selecting dimension-level responses from either the PwD or proxy, based on prior psychometric evidence identifying the most suitable respondent for each EQ-5D-5L dimension. When dyadic data are available, observed responses from the most appropriate respondent can be used. In cases in which only 1 respondent type is available, response mapping models can be applied to predict missing dimension responses, allowing a complete combined utility score to be generated.

The combined utility (integrating PwD and proxy responses) was plotted against symptom outcomes (MMSE, FAST, and PSMS scores) to evaluate the model’s predictive performance. These plots illustrate the distribution of observed versus predicted utility values across different levels of dementia symptom severity.

#### Testing pragmatic models

To ensure future applicability, additional models were estimated using the most commonly collected pragmatic variables, including PwD demographics and EQ-5D-5L reports from other respondents. The analysis results are provided in Appendix File 2 in Supplemental Materials found at https://doi.org/10.1016/j.jval.2025.05.009.

## Results

Table 1 summarizes the study data. The EPIC data set provided *n* = 765 valid EQ-5D-5L PwD self-reports (74% female), whereas the ACTIFCARE study provided *n* = 1105 valid EQ-5D-5L PwD self-reports (55% female). The EPIC sample is notably older, with more severe dementia cases (32% in severe stages [via CDR]), likely because of its focus on institutionalized populations.

**Table 1:** Summary statistics.

n (%)	ACTIFCARE	EPIC
Number of valid EQ-5D reports		
PwD	1105	765
Proxy	1182	1696
Sex of PwD		
Male	205 (45.5)	188 (26.2)
Female	246 (54.5)	529 (73.8)
Age of PwD∗		
Mean (SD)	77.8 (7.9)	85.6 (7.6)
Range	47-98	58-102
Sex of informal proxy		-
Male	151 (33.6)	
Female	299 (66.4)	
Age of informal proxy∗		-
Mean (SD)	66.4 (13.3)	
Range	25-92	
CDR stage		
0: no dementia	1 (0.1)	5 (0.3)
0.5: very mild	32 (2.7)	58 (3.5)
1: mild	779 (66.3)	367 (21.9)
2: moderate	328 (27.9)	662 (39.6)
3: severe	35 (3.0)	580 (34.7)
EQ-5D index score mean (SD)		
PwD	0.77 (0.21)	0.80 (0.23)
Proxy	0.62 (0.24)	0.62 (0.36)
Combined	0.68 (0.22)‡	0.61 (0.36)†
Proportion of EQ-5D dimension level reports by respondent type (PwD; proxy [%])		
Mobility	1: 56.3; 39.3	1: 57.6; 34.7
	2: 20.4; 24.1	2: 18.6; 14.8
	3: 16.6; 22.3	3: 9.9; 10.3
	4: 6.0; 12.3	4: 5.0; 6.9
	5: 0.7; 2.0	5: 9.0; 33.3
Self-care	1: 75.4; 43.8	1: 67.3; 13.4
	2: 13.4; 26.3	2: 16.3; 12.6
	3: 6.9; 17.4	3: 11.3; 12.9
	4: 2.3; 7.0	4: 2.9; 7.9
	5: 2.0; 5.5	5: 2.4; 53.2
Usual activities	1: 57.9; 20.8	1: 76.6; 62.6
	2: 22.5; 24.2	2: 13.8; 7.9
	3: 12.4; 27.3	3: 5.9; 9.6
	4: 5.3; 18.4	4: 1.4; 4.5
	5: 1.9: 9.4	5: 2.4; 15.4
Pain/discomfort	1: 57.2; 36.9	1: 71.9; 74.3
	2: 23.1; 31.9	2: 17.3; 15.5
	3: 15.2; 24.8	3: 7.1; 8.8
	4: 3.9; 5.5	4: 3.4; 1.2
	5: 0.6; 0.9	6: 0.4; 0.2
Anxiety/depression	1: 59.5; 38.6	1: 77.5; 75.7
	2: 26.4; 29.1	2: 14.8; 15.7
	3: 11.6; 25.4	3: 5.7; 6.7
	4: 2.2; 6.8	4: 1.4; 1.6
	5: 0.3; 0.1	5: 0.6; 0.4

*Note.* EQ-5D index score values presented in this table use matched pair observations.

CDR indicates Clinical Dementia Rating Scale; PwD; person with dementia; SD, standard deviation.

∗ At baseline.

† Combined score is statistically significantly different to PwD score (*P* < .05) but not proxy score (*P* > .05).

‡ Combined score is statistically significantly different to PwD and proxy scores (*P* < .05).

The ACTIFCARE data set reveals that the combined utility is higher than proxy values but lower than self-reported utility, with statistically significant differences observed compared with both “pure” values alone (PwD and proxy reports individually). In contrast, for the EPIC data set, the combined utility is lower than both staff proxy or PwD reports of EQ-5D-5L. The difference is statistically significant at the 5% level only in comparison with PwD-reported utilities.

Table 1 shows EQ-5D-5L dimension proportions. PwD reported lower impairment levels than proxies, especially in “mobility” and “self-care.” In EPIC, 53.2% of proxies reported the worst “self-care” level, compared with 2.4% of PwD. “Anxiety/depression” and “pain/discomfort” reports were more aligned. Across both trials, over 50% of PwD consistently reported level 1 for all dimensions, indicating ceiling effects.

Appendix Table 3 in Supplemental Materials found at https://doi.org/10.1016/j.jval.2025.05.009 explores the combined utility’s impact by CDR stage, showing consistent findings across stages. It also highlights changes in EQ-5D-5L utilities over time, with more pronounced differences between CDR stable and progressed groups from baseline to final follow-up when using the combined utility.

## Overview of Dimension Models

Variables in the final dimension models differed between data sets, reflecting distinct trial settings. In ACTIFCARE, “pain/discomfort” was predicted using proxy-assessed “anxiety/depression,” whereas EPIC used direct proxy assessments. Both trials used similar variables for “mobility” and “self-care,” and additional behavior/mood measures for “usual activities.” The final models and fit statistics demonstrate robustness (see Appendix 4 and Appendix 5 in Supplemental Materials found at https://doi.org/10.1016/j.jval.2025.05.009).

### Descriptive Margins and Model Relationships

Predictive margins plots for each dimension are provided in the Appendix File 6 in Supplemental Materials found at https://doi.org/10.1016/j.jval.2025.05.009 and illustrate the probabilities associated with each response level, providing a visual representation of the model’s predictive accuracy and highlighting areas in which the model may have limitations.

For “mobility” in ACTIFCARE, the predictive margins indicate a low probability of predicting severe impairments (level-5 reports), which corresponds with the small proportion of PwD and proxies who reported severe mobility issues (Table 1). The model’s predictions are aligned with data distributions, although it may still underrepresent the severity in cases in which severe issues are present, potentially because of the rarity of such reports in the data set.

For “self-care,” the predictive margins demonstrate that the likelihood of predicting no problems (level-1 reports) is high, particularly using ACTIFCARE data, for which the majority of PwD reported minimal difficulties (Table 1). The model struggles to predict higher levels of impairment, indicating a possible bias toward underreporting severe issues in this dimension. For ”usual activities,” the EPIC data set shows a greater probability of predicting moderate impairments (levels 3 and 4), aligning with the observed data from staff proxies.

“Pain/discomfort” and ”anxiety/depression” dimensions in both data sets reveal that models perform better at predicting lower levels of impairment (levels 1 and 2). These dimensions show a higher probability of accurately predicting mild to moderate problems, aligning well with the distribution of responses.

### Observed Versus Predicted Responses

Table 2 presents the observed versus predicted percentages of response levels for each EQ-5D-5L dimension. The models generally performed well in predicting lower severity levels across both data sets.

**Table 2:** Observed versus predicted EQ-5D-5L response frequencies.

Dimension	Response option	Observed frequency (ACTIFCARE)	Predicted frequency (ACTIFCARE)	Observed Frequency (EPIC)	Predicted frequency (EPIC)
Mobility	Level 1/No problems	56.28	56.81	34.73	41.10
	Level 2/Slight problems	20.46	20.40	14.83	18.43
	Level 3/Moderate problems	16.59	17.05	10.30	11.36
	Level 4/Severe problems	5.97	5.22	6.89	6.19
	Level 5/Unable to walk	0.70	0.52	33.25	22.92
Self-care	Level 1/No problems	43.80	47.97	13.41	20.01
	Level 2/Slight problems	26.30	26.78	12.59	19.25
	Level 3/Moderate problems	17.42	15.81	12.88	17.13
	Level 4/Severe problems	6.95	5.63	7.94	9.84
	Level 5/Unable to wash or dress	5.53	3.81	53.18	33.77
Usual activities	Level 1/No problems	20.81	23.05	62.21	70.90
	Level 2/Slight problems	24.16	24.64	7.88	11.23
	Level 3/Moderate problems	27.27	28.37	9.64	8.50
	Level 4/Severe problems	18.37	17.20	4.47	3.13
	Level 5/Unable to do usual activities	9.40	6.73	15.40	6.24
Pain/discomfort	Level 1/No pain/discomfort	57.16	57.26	71.93	71.92
	Level 2/Slight pain/discomfort	23.14	23.06	17.27	17.31
	Level 3/Moderate pain/discomfort	15.19	15.24	7.08	7.08
	Level 4/Severe pain/discomfort	3.89	3.86	3.35	3.31
	Level 5/Extreme pain/discomfort	0.62	0.58	0.37	0.38
Anxiety/depression	Level 1/No anxiety/depression	59.50	59.35	77.53	77.10
	Level 2/Slight anxiety/depression	26.44	26.14	14.77	14.92
	Level 3/Moderate anxiety/depression	11.58	12.05	5.68	5.94
	Level 4/Severe anxiety/depression	2.21	2.16	1.39	1.51
	Level 5/Extreme anxiety/depression	0.27	0.30	0.63	0.53

*Note.* Target respondent for each EQ-5D-5L dimension: mobility, PwD (ACTIFCARE), proxy (EPIC); self-care, proxy (both data sets); usual activities, proxy (both data sets); pain/discomfort, PwD (both data sets); anxiety/depression, PwD (both data sets). Therefore, the data from the opposite respondent were used to predict the target respondent’s response.

For “mobility,” the ACTIFCARE model performed reasonably well, accurately predicting both lower and moderate severity levels. Although there were some underpredictions at the highest severity levels, the model generally captured the range of mobility issues reported by PwD and proxies. In contrast, the EPIC model overestimated mid-range responses, whereas it underestimated the most severe cases. For “self-care,” both models exhibited discrepancies at extreme levels. The ACTIFCARE model was closer in predicting moderate to severe impairments but still underrepresented the highest severity. The EPIC model consistently overestimated lower severity responses, whereas it significantly underestimated severe impairments. For “usual activities,” the ACTIFCARE model aligned well with lower severity levels but underestimated higher impairments. The EPIC model overpredicted minimal impairments and underpredicted severe limitations, reflecting challenges in capturing the full range of responses in this dimension.

“Pain/discomfort” and “anxiety/depression” dimension models performed well. The dimension model for “pain/discomfort” showed strong alignment between observed and predicted frequencies at lower severity levels in both data sets. The EPIC model was particularly accurate even at higher severity levels, suggesting robustness in predicting this dimension’s responses. For “anxiety/depression,” both data set’s models performed well, particularly at lower levels. The ACTIFCARE model slightly overpredicted moderate issues, whereas the EPIC model showed minimal deviations across levels, indicating strong predictive accuracy.

Table 3 summarizes the performance of the mapping models across the EQ-5D-5L dimensions for both data sets, highlighting the strengths and limitations of the models in predicting different levels of severity, with generally strong alignment observed in ACTIFCARE compared with more challenges in severe cases within the EPIC data set. A general summary table of the key findings can be found in Appendix File 7 in Supplemental Materials found at https://doi.org/10.1016/j.jval.2025.05.009.

**Table 3:** Summary of mapping model performance across EQ-5D-5L dimensions.

Dimension	ACTIFCARE model performance	EPIC model performance	Key observations
Mobility	**✓** Accurate at predicting lower to moderate severity; **↓** slight underestimation at severe levels	**↑↓** Overestimation at mid-range; underestimation at severe levels	**✓** Strong alignment in ACTIFCARE; ✘ challenges with severe cases in EPIC
Self-care	**✓** Improved accuracy at predicting moderate to severe impairments	**↑↓** Overestimation at low severity; underestimation at severe levels	✘ Ceiling effects in PwD reports; floor effects in proxies in EPIC
Usual activities	**✓** Consistent prediction of moderate impairments; **∼** slight underestimation of severe levels	**↑↓** Overestimation of minimal impairments; underestimation of severe impairments	**✓** Model performance better in ACTIFCARE; ✘ EPIC shows significant challenges
Pain/discomfort	**✓✓** Strong alignment at lower severity levels; **∼** slight deviations at higher levels	**✓✓** Accurate predictions across most severity levels	**✓✓** Robust model performance in both data sets
Anxiety/depression	**✓** High accuracy at predicting lower levels; **↑** slight overestimation at moderate levels	**∼✓** Slight overestimation at moderate levels; good overall alignment	**✓✓** Consistent and strong model performance in both data sets

*Note.* This table summarizes the key performance metrics of mapping models across different EQ-5D-5L dimensions in the ACTIFCARE and EPIC data sets. Observations are based on predicted versus observed frequencies and key statistical metrics.

✓✓ = Highly accurate predictions.

✓ = Generally accurate predictions.

↑↓ = Overestimation and underestimation.

↓ = Underestimation.

∼ = Slight deviations in predictions.

✘= Challenges.

### Utility Distributions

**Figure 1:**
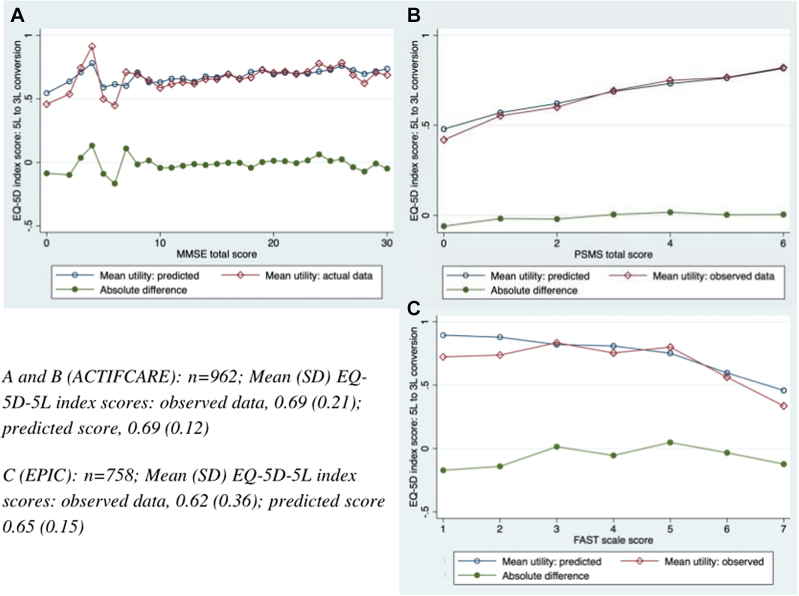
Mean predicted versus mean observed EQ-5D-5L index score over MMSE, PSMS, and FAST scores. FAST indicates Functional Assessment Staging Tool; MMSE, Mini Mental State Examination; PSMS, Personal and Social Performance Scale.

Figure 1 presents utility distributions for both data sets, comparing mean observed and predicted utilities against cognitive and functional measures (MMSE, PSMS, and FAST). For ACTIFCARE, the model generally fits well across MMSE scores, although greater variability is observed at the lower end of MMSE scores, likely due to smaller sample sizes and inherent challenges of predicting in severe cognitive impairment.

These discrepancies may stem from the low number of observations from severe impairment stages (see Appendix File 8 in Supplemental Materials found at https://doi.org/10.1016/j.jval.2025.05.009). For MMSE scores below 6, some had fewer than 4 observations, with some having only 1. Although not statistically robust, these data points were retained for transparency. Despite this, observed and predicted data showed similar distributions, with minimal prediction errors for mild to moderate cognitive impairment.

Because the EPIC trial did not collect cognition measurement, utility values were also compared with function scores (PSMS) in ACTIFCARE. The model fits well for both estimations, with only minor prediction error at lower/worse function scores. Some of the lower PSMS scores had few observations, contributing to variability in those estimates.

In the EPIC data set, the utility values were plotted against FAST scores (Fig. 1), revealing that the model overestimated utility values at both extremes of FAST scores. Similarly, there were very few observations for individuals in the most severe FAST stages. The number of utility value estimations were highest for FAST scale scores of 4 and 6 (4, *n* = 114; 6, *n* = 510), with these levels reflecting moderate dementia. Low numbers of observations in these stages contribute to observed prediction error, which was minimized for individuals with mild to moderate functional impairment, indicating that the model performed best within this subgroup.

Table 4 compares mean EQ-5D-5L utilities for the observed and predicted data across the United Kingdom, Australia, United States, and The Netherlands value sets. Appendix 9 in Supplemental Materials found at https://doi.org/10.1016/j.jval.2025.05.009 presents the corresponding utility distribution plots. These additional analyses did not reveal any new or noteworthy patterns beyond those identified in the primary analysis.

**Table 4:** EQ-5D-5L value set comparisons.

Mean, SD	UK	Australia	The Netherlands	USA
ACTIFCARE*, n = 962*				
Observed	0.77 (0.18)	0.81 (0.20)	0.72 (0.19)	0.70 (0.23)
Predicted	0.77 (0.10)	0.80 (0.12)	0.72 (0.10)	0.70 (0.14)
EPIC*, n = 758*				
Observed	0.77 (0.19)	0.72 (0.24)	0.82 (0.18)	0.82 (0.23)
Predicted	0.72 (0.09)	0.67 (0.12)	0.71 (0.08)	0.65 (0.12)

*Note.* Table shows observed and predicted EQ-5D-5L utility scores when applying different country value sets.

### Pragmatic Models

Pragmatic models revealed that it is possible to predict EQ-5D-5L dimension levels using only the selected pragmatic variables, indicating that these simplified models do have some utility. However, when compared with more extensive models that include additional symptom variable scores, the performance of the pragmatic models was noticeably poorer. Specifically, the predicted utility value distributions from the pragmatic models showed greater error, highlighting the trade-off between model simplicity and predictive accuracy (see Appendix File 2 in Supplemental Materials found at https://doi.org/10.1016/j.jval.2025.05.009 for analysis output).

## Discussion

This study aimed to assess the feasibility and accuracy of generating combined EQ-5D-5L utilities using statistical mapping techniques when data from only 1 respondent type (either PwD or proxy) is available. By using data from the ACTIFCARE and EPIC studies, this analysis offers insights into the potential application of these combined utilities in economic evaluations of dementia interventions, particularly in situations in which dyadic respondent data are not feasible. HRQoL levels are often assigned based on dementia severity stage in economic models, using data from general population preference panels, mapped instruments, or patient and caregiver interviews. Our approach contributes to this by providing a method to generate combined utility values when only 1 respondent type is available, offering an alternative way to estimate HRQoL inputs for economic models. This study provides a methodological approach to integrating utility scores by leveraging observed dyadic data where available and applying mapping techniques when only one respondent’s data are collected. Although mapping introduces some error, it allows combined scores to be generated even in the absence of full dyadic data.

### Impact on Utility Values

By combining reports, utility values provide a more accurate representation of the PwD’s health state, enhancing the sensitivity of quality-adjusted life-year calculations. This increased sensitivity makes it more likely to detect subtle changes in health status resulting from treatments, whether these reflect benefits or the absence of benefits, thereby potentially optimizing the evaluation of intervention outcomes. In ACTIFCARE, the combined utilities fell between PwD self-reports and proxy assessments, integrating 2 proxy-reported dimensions. This closer alignment with proxy ratings in EPIC may reflect the study’s setting and respondent composition. EPIC included institutionalized PwD with more severe dementia, in which proxies were professional caregivers, who tend to report lower HRQoL than informal caregivers in ACTIFCARE. Additionally, the greater reliance on proxy-reported dimensions in EPIC likely amplified this effect. The large discrepancy between the number of PwD reports (*n* = 765) and proxy reports (*n* = 1696) in EPIC reflects the challenges of collecting self-reported data in severe dementia. This difference affects the results by reducing the number of dyads available for direct comparisons and increasing the reliance on proxy data in severe cases. In contrast, the EPIC combined utilities were lower than both “pure” utilities, likely because it included 3 proxy-reported dimensions. This reflects the inclusion of dimensions most sensitive to change from the target respondent, making the combined utility more likely to detect meaningful health status changes, which is the goal when evaluating treatment effects. However, the accuracy of these utilities depends on the reliability of dimension-level predictions. When predictions deviate from observed data, there is a risk of introducing errors into overall utility, particularly in dimensions with significant ceiling/floor effects.

### Mapping Model Performance

Ordered probit models produced utility predictions that aligned well with observed data, particularly for ACTIFCARE. The models performed effectively for mild-to-moderate dementia, minimizing prediction errors. Accuracy was somewhat reduced at extreme dementia severities, particularly in the EPIC data set, including institutionalized populations, likely reflecting limited numbers of observations in these categories. Despite this observation, data points were retained for transparency. Overall, the models performed strongly, and further testing in data sets with greater numbers of severe cases could enhance their applicability across dementia severity.

### Dimension-Level Differences

The mapping models’ performance varied by data and dementia severity. Table 3 compares predictive accuracy across dimensions. “Pain/discomfort” and “anxiety/depression” showed strong alignment between predicted and observed data, suggesting robust model performance. Notably, these dimensions used proxy reports to predict PwD reports, indicating that proxy data can be reliably used to predict PwD responses in certain dimensions. This finding highlights the potential advantage in collecting proxy reports because they can be used to accurately estimate the PwD’s reports for dimensions for which PwD is the target respondent. However, predicting severe impairments in “mobility” and “self-care” posed challenges, particularly in EPIC, in which ceiling and floor effects likely contributed. Context, such as dementia severity and residential setting, significantly affect model performance; and models are limited by underlying data distributions, in which ceiling and floor effects can limit precision.

### Utility of Pragmatic Models

The pragmatic models used a reduced set of variables and demonstrated that it is possible to predict EQ-5D-5L dimension responses with simplified models. However, these models showed reduced accuracy compared with the more detailed models that included additional symptom variables. This trade-off between simplicity and predictive accuracy suggests that although pragmatic models may be useful in certain settings, they should be applied with caution. Future dementia trials should focus on collecting unified outcomes to enhance the reliability of this type of analysis. Core outcome sets for dementia studies already exist, categorized by the type of intervention under investigation, and were summarized in our previous article,^2^ which served as a precursor to this work. Collecting standardized outcomes not only enhances consistency across studies but also ensures that trialists gather the necessary data to support retrospective economic evaluations. This forward-thinking approach enables mapping algorithms such as ours to be applied in future analyses, even if a trial was not initially designed for economic evaluation.

### Application of Different Country Value Sets

Utility values were also estimated using value sets from other countries^19^, ^20^, ^21^ and did not reveal any new patterns beyond those in the primary analysis but emphasized the sensitivity of utility estimates to the choice of value set. This finding highlights the importance of carefully selecting the appropriate value set for utility estimation because this can significantly influence the outcomes of economic evaluations and affect health policy decisions.

### Strengths and Limitations

This study innovatively uses existing data from 2 well-established dementia trials, providing a valuable contribution to the field by addressing the longstanding challenge of poor interrater agreement between PwD and proxies. The robust and transparent methodological approach, including the iterative process of model development and refinement, further strengthens the validity of the findings.

However, the reliance on only 2 data sets limits the generalizability of the results, and the inability to perform out-of-sample validation is a significant constraint. The focus on response mapping without comparing it with other mapping techniques restricts the scope of the analysis. Differences in proxy perspectives also introduce variability because informal caregivers may focus on emotional well-being, whereas professional caregivers emphasize functional limitations, potentially biasing utility estimates. Future work should explore whether adjusting for caregiver burden, where data permit, could improve the accuracy and fairness of these approaches. Additionally, dementia is characterized by health fluctuations, which may affect HRQoL assessments using “today” recall periods because momentary changes in cognition and behavior can influence self- and proxy-reported scores.^22^ EQ-5D’s lack of a cognition dimension and its susceptibility to ceiling effects in mild dementia may further limit its sensitivity to HRQoL changes in dementia populations. Another challenge relates to EQ-5D’s dimensions. PwD may underestimate objective aspects, such as ADLs, because of impaired insight, whereas subjective dimensions such as pain and anxiety are more reliably self-reported in mild to moderate stages. In severe dementia, self-reporting becomes less feasible, and observational scales may be needed for more accurate pain and anxiety assessment. Finally, the challenges in accurately predicting utility values for severe dementia and the differences in model performance across dimensions and data sets highlight the need for high-quality data collection spanning the dementia severity range and model refinement.

## Conclusions

This study demonstrates the feasibility of predicting EQ-5D-5L dimension responses and generates combined utility values using data from a single respondent type. Although the models performed well for mild-to-moderate dementia, the data availability for severe dementia was insufficient to draw strong conclusions. Further refinement is needed to extend these findings to severe dementia and to better capture the variability in different residential settings. A combined utility approach offers a promising tool for enhancing the accuracy of HRQoL assessments in dementia, with potential applications in economic evaluations that could lead to better informed healthcare decisions and policies. Future research should focus on validating these models in diverse populations, exploring alternative mapping techniques and prioritizing high-quality, complete data collection.

## Article and Author Information

**Authorship Confirmation:** All authors certify that they meet the ICMJE criteria for authorship.

**Funding/Support:** This research was undertaken as part of a 10.13039/501100000858University of Sheffield studentship relating to the National Institute for Health and Care Research's 10.13039/100024348Policy Research Unit in Economic Methods of Evaluation in 10.13039/100017291Health and Social Care Interventions (EEPRU), PR-PRU-1217-20401.

**Role of the Funder/Sponsor:** The funder had no role in the design and conduct of the research, analysis, or interpretation of the data, nor in the preparation, review, or approval of the manuscript. Although the funder had the right to provide comments, there was no obligation for us to consider or implement them in the manuscript.

## Author Disclosures

Author disclosure forms can be accessed below in the Supplemental Material section. Dr Wailoo and Dr Rowen are editors for *Value in Health* and had no role in the peer-review process of this article.

## Supplemental Material

Author Disclosures

Appendices
