# Supplementary material for: Mapping EQ-5D-5L Utilities in Dementia: Integrating Self and Proxy Reports
Source: Value Health. 2025 Aug;28(8):1204–12. doi: 10.1016/j.jval.2025.05.009 (PMC12311261; doi:10.1016/j.jval.2025.05.009)
Supplement: Author Disclosures [file mmc1.pdf]

Hannah Hussain

Discloser Identifier: 125753885

Disclosure Purpose: VIH-2024-1119

Employment Information: Currently Employed

Summary of Interests

Company or Organization

Entity	Type	Relevant to this Disclosure
The Office of Health Economics	Consultant Current Employment	
**Category:** Consultant		
University of Sheffield	Grant / Contract	Yes

Additional Questions

1. **Please select which of the following apply to each relationship or activity:**
   a. **Other Professional Activities - Consultant** The Office of Health Economics

      Neither

   b. **Grant / Contract** University of Sheffield

      The relationship is in direct support of the work reported in the manuscript anytime from when the work was conceived

2. **I confirm I have disclosed all direct support for the present manuscript (e.g. funding, provision of study materials, medical writing, article processing charges, etc.) There is no time limit for this item.**

   Yes

3. **Please indicate below whether in the past 36 months you have had any of the following interests that are topically related to the work reported in the manuscript.**
   a. **Employment (If you need to add an interest, please scroll to the top of the page, click "add interest" and select "Employment")**

      Yes, as disclosed above

   b. **Grants or contracts for research (If you need to add an interest, please scroll to the top of the page, click "add interest" and select "Grant/Contract")**

      Yes, as disclosed above

   c. **Payment for consulting (If you need to add an interest, please scroll to the top of the page, click "add interest" and select "Independent Contractor")**

      No, I have no relevant interests of this type

   d. **Payments or honoraria for lectures, presentations, speakers bureaus, or educational events (If you need to add an interest, please scroll to the top of the page, click "add interest" and select "Independent Contractor" and include the correct information under "Consultant")**

      No, I have no relevant interests of this type

e. **Payment for service on an advisory board (If you need to add an interest, please scroll to the top of the page, click "add interest" and select "Independent Contractor," and choose "Other")**

No, I have no relevant interests of this type

f. **Payment for participation Data and safety monitoring board (If you need to add an interest, please scroll to the top of the page, click "add interest" and select "Independent Contractor")**

No, I have no relevant interests of this type

g. **Expert witness testimony (If you need to add an interest, please scroll to the top of the page, click "add interest" and select "Independent Contractor")**

No, I have no relevant interests of this type

h. **Royalties from Patents, Trademarks, Copyrights or other intellectual property (If you need to add an interest, please scroll to the top of the page, click "add interest" and select the appropriate interest type)**

No, I have no relevant interests of this type

i. **Patents planned, issued, or pending, whether or not you receive royalties (If you need to add an interest, please scroll to the top of the page, click "add interest" and select "Patents")**

No, I have no relevant interests of this type

j. **Fiduciary Officer or Other Board Membership (If you need to add an interest, please scroll to the top of the page, click "add interest" and select "Fiduciary Officer")**

No, I have no relevant interests of this type

k. **Stock or stock options (If you need to add an interest, please scroll to the top of the page, click "add interest" and select the appropriate interest type)**

No, I have no relevant interests of this type

l. **Support for attending meetings or other travel (If you need to add an interest, please scroll to the top of the page, click "add interest" and select "Travel")**

No, I have no relevant interests of this type

4. **Was any individual paid to provide professional writing assistance with this manuscript?**

   No.

5. **Have you or your institution received equipment, materials, drugs, or services in direct support of the work in the manuscript (without time limit) not disclosed above?**

   No.

6. **In the past 36 months, have you received equipment, materials, drugs, medical writing, gifts or other services from for-profit or not-for-profit third parties whose interests may be affected by the content of the manuscript not disclosed above?**

   No.

7. **Are there other financial or non-financial interests that readers could perceive to have influenced, or that give the appearance of potentially influencing, what you wrote in the submitted work not disclosed above.**

   No.

Certification

I certify that I have answered every question and the information provided in this disclosure is complete and accurate.

Anju Keetharuth

Discloser Identifier: 55026472

Disclosure Purpose: VIH-2024-1119

Employment Information: Currently Employed

Summary of Interests

I do not have any interests to disclose at this time.

Additional Questions

1. **Please select which of the following apply to each relationship or activity:**

   You are not disclosing any interests to this organization.

2. **I confirm I have disclosed all direct support for the present manuscript (e.g. funding, provision of study materials, medical writing, article processing charges, etc.) There is no time limit for this item.**

   Yes

3. **Please indicate below whether in the past 36 months you have had any of the following interests that are topically related to the work reported in the manuscript.**

   a. **Employment (If you need to add an interest, please scroll to the top of the page, click "add interest" and select "Employment")**

      No, I have no relevant interests of this type

   b. **Grants or contracts for research (If you need to add an interest, please scroll to the top of the page, click "add interest" and select "Grant/Contract")**

      No, I have no relevant interests of this type

   c. **Payment for consulting (If you need to add an interest, please scroll to the top of the page, click "add interest" and select "Independent Contractor")**

      No, I have no relevant interests of this type

   d. **Payments or honoraria for lectures, presentations, speakers bureaus, or educational events (If you need to add an interest, please scroll to the top of the page, click "add interest" and select "Independent Contractor" and include the correct information under "Consultant")**

      No, I have no relevant interests of this type

   e. **Payment for service on an advisory board (If you need to add an interest, please scroll to the top of the page, click "add interest" and select "Independent Contractor," and choose "Other")**

      No, I have no relevant interests of this type

   f. **Payment for participation Data and safety monitoring board (If you need to add an interest, please scroll to the top of the page, click "add interest" and select "Independent Contractor")**

      No, I have no relevant interests of this type

   g. **Expert witness testimony (If you need to add an interest, please scroll to the top of the page, click "add interest" and select "Independent Contractor")**

      No, I have no relevant interests of this type

   h. **Royalties from Patents, Trademarks, Copyrights or other intellectual property (If you need to add an interest, please scroll to the top of the page, click "add interest" and select the appropriate interest type)**

No, I have no relevant interests of this type

i. **Patents planned, issued, or pending, whether or not you receive royalties (If you need to add an interest, please scroll to the top of the page, click "add interest" and select "Patents")**

No, I have no relevant interests of this type

j. **Fiduciary Officer or Other Board Membership (If you need to add an interest, please scroll to the top of the page, click "add interest" and select "Fiduciary Officer")**

No, I have no relevant interests of this type

k. **Stock or stock options (If you need to add an interest, please scroll to the top of the page, click "add interest" and select the appropriate interest type)**

No, I have no relevant interests of this type

l. **Support for attending meetings or other travel (If you need to add an interest, please scroll to the top of the page, click "add interest" and select "Travel")**

No, I have no relevant interests of this type

4. **Was any individual paid to provide professional writing assistance with this manuscript?**

No.

5. **Have you or your institution received equipment, materials, drugs, or services in direct support of the work in the manuscript (without time limit) not disclosed above?**

No.

6. **In the past 36 months, have you received equipment, materials, drugs, medical writing, gifts or other services from for-profit or not-for-profit third parties whose interests may be affected by the content of the manuscript not disclosed above?**

No.

7. **Are there other financial or non-financial interests that readers could perceive to have influenced, or that give the appearance of potentially influencing, what you wrote in the submitted work not disclosed above.**

No.

Certification

I certify that I have answered every question and the information provided in this disclosure is complete and accurate.

Donna Rowen

Discloser Identifier: 55032238

Disclosure Purpose: VIH-2024-1119

Employment Information: Currently Employed

Summary of Interests

Company or Organization

Entity	Type	Relevant to this Disclosure
EuroQol Research Foundation	Other	Yes
**Category:** Other		
EuroQol Research Foundation	Travel	Yes
Horizon 2020 Framework Programme	Grant / Contract	
Innovative Medicines Initiative 2 Joint Undertaking	Grant / Contract	
Innovative Medicines Initiative 2 Joint Undertaking	Grant / Contract	
University of Sheffield	Employment Current Employment	Yes
**Title:** Professor of Helath Economics		

Additional Questions

1. **Please select which of the following apply to each relationship or activity:**
   a. **Other Professional Activities - Other** EuroQol Research Foundation

      The relationship is outside the work reported in the manuscript but topically related and within the past 36 months

   b. **Employment** University of Sheffield

      The relationship is in direct support of the work reported in the manuscript anytime from when the work was conceived

   c. **Grant / Contract** Horizon 2020 Framework Programme

      Neither

   d. **Grant / Contract** Innovative Medicines Initiative 2 Joint Undertaking

      Neither

   e. **Grant / Contract** Innovative Medicines Initiative 2 Joint Undertaking

      Neither

   f. **Travel** EuroQol Research Foundation

      The relationship is outside the work reported in the manuscript but topically related and within the past 36 months

2. **I confirm I have disclosed all direct support for the present manuscript (e.g. funding, provision of study materials, medical writing, article processing charges, etc.) There is no time limit for this item.**

   Yes

3. **Please indicate below whether in the past 36 months you have had any of the following interests that are topically related to the work reported in the manuscript.**

   a. **Employment (If you need to add an interest, please scroll to the top of the page, click "add interest" and select “Employment”)**

      Yes, as disclosed above

   b. **Grants or contracts for research (If you need to add an interest, please scroll to the top of the page, click "add interest" and select “Grant/Contract”)**

      Yes, as disclosed above

   c. **Payment for consulting (If you need to add an interest, please scroll to the top of the page, click "add interest" and select “Independent Contractor”)**

      No, I have no relevant interests of this type

   d. **Payments or honoraria for lectures, presentations, speakers bureaus, or educational events (If you need to add an interest, please scroll to the top of the page, click "add interest" and select "Independent Contractor" and include the correct information under "Consultant")**

      No, I have no relevant interests of this type

   e. **Payment for service on an advisory board (If you need to add an interest, please scroll to the top of the page, click "add interest" and select “Independent Contractor,” and choose “Other”)**

      No, I have no relevant interests of this type

   f. **Payment for participation Data and safety monitoring board (If you need to add an interest, please scroll to the top of the page, click "add interest" and select “Independent Contractor”)**

      No, I have no relevant interests of this type

   g. **Expert witness testimony (If you need to add an interest, please scroll to the top of the page, click "add interest" and select “Independent Contractor”)**

      No, I have no relevant interests of this type

   h. **Royalties from Patents, Trademarks, Copyrights or other intellectual property (If you need to add an interest, please scroll to the top of the page, click "add interest" and select the appropriate interest type)**

      No, I have no relevant interests of this type

   i. **Patents planned, issued, or pending, whether or not you receive royalties (If you need to add an interest, please scroll to the top of the page, click "add interest" and select “Patents”)**

      No, I have no relevant interests of this type

   j. **Fiduciary Officer or Other Board Membership (If you need to add an interest, please scroll to the top of the page, click "add interest" and select “Fiduciary Officer”)**

      No, I have no relevant interests of this type

   k. **Stock or stock options (If you need to add an interest, please scroll to the top of the page, click "add interest" and select the appropriate interest type)**

      No, I have no relevant interests of this type

   l. **Support for attending meetings or other travel (If you need to add an interest, please scroll to the top of the page, click "add interest" and select “Travel”)**

      Yes, as disclosed above

4. **Was any individual paid to provide professional writing assistance with this manuscript?**

   No.

5. **Have you or your institution received equipment, materials, drugs, or services in direct support of the work in the manuscript (without time limit) not disclosed above?**

No.

6. **In the past 36 months, have you received equipment, materials, drugs, medical writing, gifts or other services from for-profit or not-for-profit third parties whose interests may be affected by the content of the manuscript not disclosed above?**

   No.

7. **Are there other financial or non-financial interests that readers could perceive to have influenced, or that give the appearance of potentially influencing, what you wrote in the submitted work not disclosed above.**

   No.

Certification

I certify that I have answered every question and the information provided in this disclosure is complete and accurate.

Convey Global Disclosure System AAMC

Allan Wailoo

Discloser Identifier: 55105698

Disclosure Purpose: VIH-2024-1119

Employment Information: Currently Employed

Summary of Interests

I do not have any interests to disclose at this time.

Additional Questions

1. **Please select which of the following apply to each relationship or activity:**

   You are not disclosing any interests to this organization.

2. **I confirm I have disclosed all direct support for the present manuscript (e.g. funding, provision of study materials, medical writing, article processing charges, etc.) There is no time limit for this item.**

   Yes

3. **Please indicate below whether in the past 36 months you have had any of the following interests that are topically related to the work reported in the manuscript.**

   a. **Employment (If you need to add an interest, please scroll to the top of the page, click "add interest" and select “Employment”)**

      No, I have no relevant interests of this type

   b. **Grants or contracts for research (If you need to add an interest, please scroll to the top of the page, click "add interest" and select “Grant/Contract”)**

      Yes, as disclosed above

   c. **Payment for consulting (If you need to add an interest, please scroll to the top of the page, click "add interest" and select “Independent Contractor”)**

      No, I have no relevant interests of this type

   d. **Payments or honoraria for lectures, presentations, speakers bureaus, or educational events (If you need to add an interest, please scroll to the top of the page, click "add interest" and select "Independent Contractor" and include the correct information under "Consultant")**

      No, I have no relevant interests of this type

   e. **Payment for service on an advisory board (If you need to add an interest, please scroll to the top of the page, click "add interest" and select “Independent Contractor,” and choose “Other”)**

      No, I have no relevant interests of this type

   f. **Payment for participation Data and safety monitoring board (If you need to add an interest, please scroll to the top of the page, click "add interest" and select “Independent Contractor”)**

      No, I have no relevant interests of this type

   g. **Expert witness testimony (If you need to add an interest, please scroll to the top of the page, click "add interest" and select “Independent Contractor”)**

      No, I have no relevant interests of this type

   h. **Royalties from Patents, Trademarks, Copyrights or other intellectual property (If you need to add an interest, please scroll to the top of the page, click "add interest" and select the appropriate interest type)**

No, I have no relevant interests of this type

i. **Patents planned, issued, or pending, whether or not you receive royalties (If you need to add an interest, please scroll to the top of the page, click "add interest" and select “Patents”)**

No, I have no relevant interests of this type

j. **Fiduciary Officer or Other Board Membership (If you need to add an interest, please scroll to the top of the page, click "add interest" and select “Fiduciary Officer”)**

No, I have no relevant interests of this type

k. **Stock or stock options (If you need to add an interest, please scroll to the top of the page, click "add interest" and select the appropriate interest type)**

No, I have no relevant interests of this type

l. **Support for attending meetings or other travel (If you need to add an interest, please scroll to the top of the page, click "add interest" and select “Travel”)**

No, I have no relevant interests of this type

4. **Was any individual paid to provide professional writing assistance with this manuscript?**

No.

5. **Have you or your institution received equipment, materials, drugs, or services in direct support of the work in the manuscript (without time limit) not disclosed above?**

No.

6. **In the past 36 months, have you received equipment, materials, drugs, medical writing, gifts or other services from for-profit or not-for-profit third parties whose interests may be affected by the content of the manuscript not disclosed above?**

No.

7. **Are there other financial or non-financial interests that readers could perceive to have influenced, or that give the appearance of potentially influencing, what you wrote in the submitted work not disclosed above.**

No.

Certification

I certify that I have answered every question and the information provided in this disclosure is complete and accurate.

Convey Global Disclosure System | AAMC
