# Supplementary material for: Mapping EQ-5D-5L Utilities in Dementia: Integrating Self and Proxy Reports
Source: Value Health. 2025 Aug;28(8):1204–12. doi: 10.1016/j.jval.2025.05.009 (PMC12311261; doi:10.1016/j.jval.2025.05.009)

**Appendix 1**

**Summary of previous analysis: assessing EQ-5D responses by dimension**

Our previous analyses evaluated the effectiveness of EQ-5D responses collected from PwD and their proxies. This analysis was informed by our previous systematic review that assessed EQ-5D’s convergent validity with dementia outcomes, identifying relevant dementia symptoms for comparison against EQ-5D dimensions {Hussain, 2022 #270}. The analyses used data from three dementia trials, ACTIFCARE, EPIC and REMCARE, to assess the performance and accuracy of EQ-5D dimension reports in reflecting changes in dementia symptomatology. The objectives of this work included identifying which respondent type (PwD or proxy) provides the most accurate EQ-5D dimension reports and making recommendations for combining these reports to generate a hybrid/combined utility score for use in dementia economic evaluations.

**Objectives**

1. **Determine Accurate Respondents:** The analysis aimed to identify whose EQ-5D dimension reports (PwD or proxy) most accurately reflect changes in dementia symptoms.
2. **Inter-Rater Agreement Analysis:** It investigated similarities and differences between PwD and proxy reports using inter-rater agreement methods, including Kappa coefficients and exact agreement percentages.
3. **Recommendations for Dimension Reporting:** Based on empirical evidence, guidance on which respondent's reports to use for each EQ-5D dimension, especially in calculating utility scores for economic evaluations of dementia interventions was determined.

**Methods**

The analyses relied on individual patient-level data and applied statistical and psychometric techniques to assess the alignment of EQ-5D dimension reports with external dementia symptom measures. The studies differ in setting and severity stages, with ACTIFCARE focusing on community-dwelling PwD and EPIC on institutionalised PwD. The methods used in the analyses included:

- **Dyadic Change Analysis:** This approach assesses changes in EQ-5D reports from both PwD and proxies over time, focusing on the directions of change (improvement, worsening, or no change) relative to dementia symptom progression.
- **Responsiveness Analysis:** Responsiveness was assessed by calculating effect sizes (using Cohen’s d) to determine how well EQ-5D dimension scores captured changes in dementia symptoms over time. This analysis helped identify which dimension reports were most sensitive to changes in symptom severity.
- **Inter-Rater Agreement:** The level of agreement between PwD and proxy reports was measured using Kappa coefficients and intra-class correlation coefficients (ICCs). Bland-Altman plots were used to visually assess agreement levels.
- **Discrepancy Scores:** Absolute discrepancy scores were calculated to evaluate differences in dimension reporting between PwD and proxies, highlighting the degree of agreement and any potential biases in reporting.
- **Floor and Ceiling Effects:** The analyses assessed the proportion of respondents reporting either the highest or lowest scores across all EQ-5D dimensions to determine any limitations in response variability.

**Key Findings**

- **Community vs. Institutional Settings:** For community-dwelling PwD, informal proxies (such as family members or friends) are recommended because they provide valuable insights into the individual's health status in a personal context. In institutional settings, staff proxies are preferred due to their professional training and regular interactions with residents. This approach is pragmatic, as missing data is higher for informal proxies in institutional settings, whereas staff proxy data is virtually complete. Additionally, collecting three sources of data about one respondent may be unnecessary.
- **Mobility Dimension:** Staff proxy reports were more responsive to changes in dementia symptoms compared to PwD self-reports and informal proxies, especially in institutionalised settings.
- **Self-Care Dimension:** Proxy reports, particularly from staff in institutional settings, provided more reliable data than PwD self-reports, which exhibited significant ceiling effects.
- **Usual Activities Dimension:** Both proxy types were preferred over PwD self-reports due to substantial ceiling effects observed in self-reports.
- **Pain/Discomfort and Anxiety/Depression Dimensions:** PwD self-reports were retained as the preferred source of data due to the subjective nature of these dimensions and the lack of substantial evidence to support proxy reports over self-reports.

Table A1) EQ-5D target dimension reports by respondent and residential status of PwD

|  | **Residential setting of PwD** | |
| --- | --- | --- |
| **EQ-5D dimension** | *Community dwelling* | *Institutionalised* |
| Mobility | PwD | Staff proxy |
| Self-care | Informal proxy | Staff proxy |
| Usual activities | Informal proxy | Staff proxy |
| Pain/ discomfort | PwD | PwD |
| Anxiety/ depression | PwD | PwD |

**Conclusion**

This analysis concludes that a mixed approach, utilising both PwD and proxy reports, may be optimal for accurately assessing HRQoL in dementia studies. For some dimensions, proxies provide more accurate data, especially in institutional settings, while for others, PwD self-reports are more appropriate. This nuanced approach allows for a more accurate reflection of the PwD's condition in economic evaluations, potentially impacting decision-making processes regarding dementia care interventions.

**Appendix 2**

**Pragmatic model generation – ACTIFCARE**

**Mobility**

*oprobit eq1SR_ i.eq1RP_ PwD_age_ i.PwDs_sex, vce(cluster PatNo)*

| Response options | Observed frequency (%)  *n=1139* | Predicted frequency (%) |
| --- | --- | --- |
| No problems | 56.28 | 55.63 |
| Slight problems | 20.46 | 20.22 |
| Moderate problems | 16.59 | 16.65 |
| Severe problems | 5.97 | 6.51 |
| Unable to walk | 0.70 | 0.99 |

**Self-care**

*oprobit eq2RP_ i.eq2SR_ PwD_age_ i.PwDs_sex_, vce(cluster PatNo)*

| Response options | Observed frequency (%)  *n=1194* | Predicted frequency (%) |
| --- | --- | --- |
| No problems | 43.80 | 46.18 |
| Slight problems | 26.30 | 27.02 |
| Moderate problems | 17.42 | 16.47 |
| Severe problems | 6.95 | 6.03 |
| Unable to wash or dress | 5.53 | 4.30 |

**Usual activities**

*oprobit eq3RP_ i.eq3SR_ PwD_age_ i.PwDs_sex_, vce(cluster PatNo)*

| Response options | Observed frequency (%)  *n=1192* | Predicted frequency (%) |
| --- | --- | --- |
| No problems | 20.81 | 22.17 |
| Slight problems | 24.16 | 25.02 |
| Moderate problems | 27.27 | 27.82 |
| Severe problems | 18.37 | 17.57 |
| Unable to do usual activities | 9.40 | 7.42 |

**Pain/discomfort**

*oprobit eq4RP_ i.eq5RP_ PwD_age_ i.PwDs_sex_, vce(cluster PatNo)*

| Response options | Observed frequency (%)  *n=1132* | Predicted frequency (%) |
| --- | --- | --- |
| No pain/discomfort | 57.16 | 57.04 |
| Slight pain/discomfort | 23.14 | 23.37 |
| Moderate pain/discomfort | 15.19 | 14.94 |
| Severe pain/discomfort | 3.89 | 4.13 |
| Extreme pain/discomfort | 0.62 | 0.52 |

**Anxiety/depression**

*oprobit eq5SR_ i.eq5RP PwD_age_ i.PwDs_sex_, vce(cluster PatNo)*

| Response options | Observed frequency (%)  *n=1131* | Predicted frequency (%) |
| --- | --- | --- |
| No anxiety/depression | 59.50 | 59.75 |
| Slight anxiety/depression | 26.44 | 26.10 |
| Moderate anxiety/depression | 11.58 | 11.70 |
| Severe anxiety/depression | 2.21 | 2.18 |
| Extreme anxiety/depression | 0.27 | 0.27 |

| Target response | N | Mean | SD | Min – Max |
| --- | --- | --- | --- | --- |
| Mapped from 5L to 3L value set | | | | |
| Observed data | 962 | 0.69 | 0.21 | -0.28 – 0.99 |
| Predicted data | 962 | 0.67 | 0.09 | 0.14 – 0.82 |


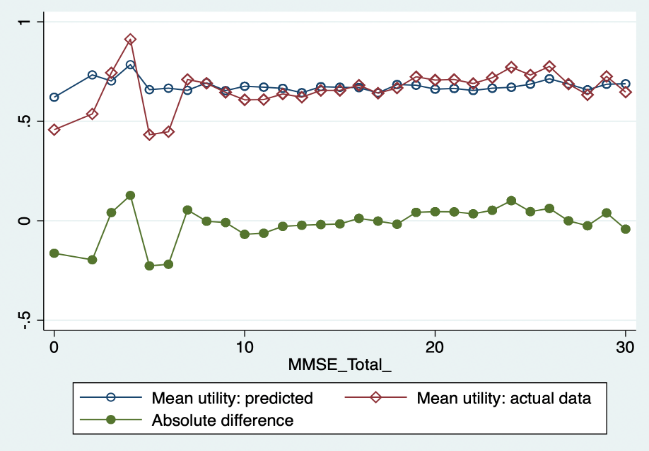

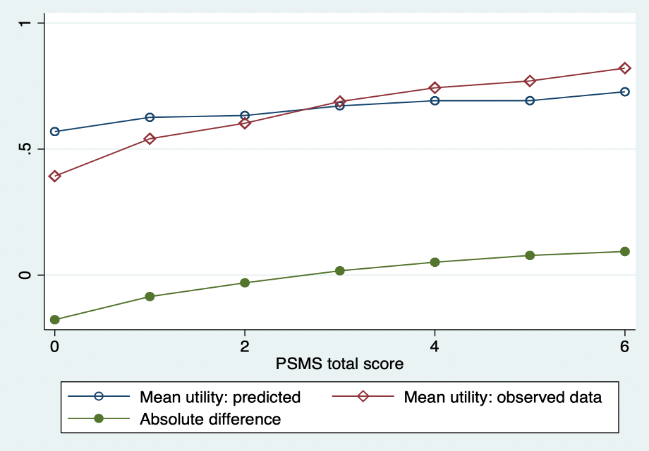


**Pragmatic model generation – EPIC**

**Mobility**

*oprobit eq1staff_ i.eq1SR_ age_ i.sex_, vce(cluster PatNo)*

| Mobility response level | Observed frequency (%)  n=1699 | Predicted frequency (%) |
| --- | --- | --- |
| 1 | 34.73 | 41.15 |
| 2 | 14.83 | 18.59 |
| 3 | 10.30 | 11.13 |
| 4 | 6.89 | 6.13 |
| 5 | 33.25 | 23.00 |

**Self-care**

*oprobit eq2staff_ i.eq2SR_ age_ i.sex_, vce(cluster PatNo)*

| Mobility response level | Observed frequency (%)  n=1700 | Predicted frequency (%) |
| --- | --- | --- |
| 1 | 13.41 | 20.13 |
| 2 | 12.59 | 19.58 |
| 3 | 12.88 | 17.02 |
| 4 | 7.94 | 9.66 |
| 5 | 53.18 | 33.61 |

**Usual activities**

*oprobit eq3staff_ i.eq3SR_ age_ i.sex_, vce(cluster PatNo)*

| Response options | Observed frequency (%)  *n=1701* | Predicted frequency (%) |
| --- | --- | --- |
| No problems | 62.21 | 71.06 |
| Slight problems | 7.88 | 11.13 |
| Moderate problems | 9.64 | 8.35 |
| Severe problems | 4.47 | 3.07 |
| Unable to do usual activities | 15.40 | 6.39 |

**Pain/discomfort**

*oprobit eq4SR_ eq4staff i.sex_ age_, vce(cluster PatNo)*

| Response options | Observed frequency (%)  *n=805* | Predicted frequency (%) |
| --- | --- | --- |
| No pain/discomfort | 71.93 | 71.92 |
| Slight pain/discomfort | 17.27 | 17.31 |
| Moderate pain/discomfort | 7.08 | 7.07 |
| Severe pain/discomfort | 3.35 | 3.31 |
| Extreme pain/discomfort | 0.37 | 0.38 |

**Anxiety/depression**

*oprobit eq5SR_ i.eq5staff_ i.sex_ age_, vce(cluster PatNo)*

| Response options | Observed frequency (%)  *n=792* | Predicted frequency (%) |
| --- | --- | --- |
| No anxiety/depression | 77.53 | 77.48 |
| Slight anxiety/depression | 14.77 | 14.71 |
| Moderate anxiety/depression | 5.68 | 5.78 |
| Severe anxiety/depression | 1.39 | 1.41 |
| Extreme anxiety/depression | 0.63 | 0.62 |

| Target response | N | Mean |
| --- | --- | --- |
| Mapped from 5L to 3L value set | | |
| Observed data | 758 | 0.62 |
| Predicted data | 758 | 0.65 |


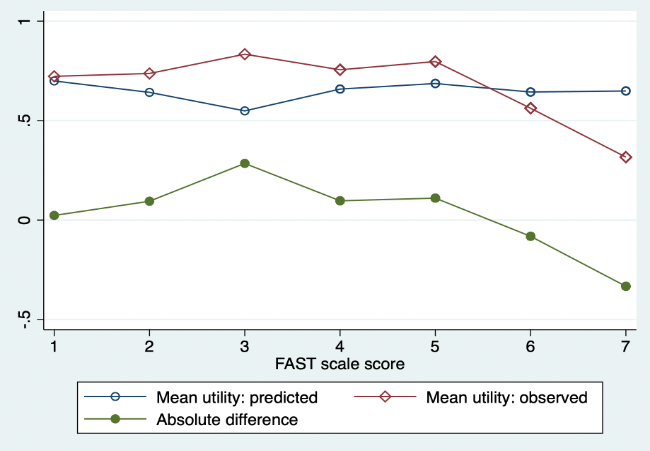


**Appendix 3**

Table A3) Mean EQ-5D by respondent type and disease severity stage

| **Severity stage, ACTIFCARE** | **PwD** | **Relative Proxy** | **Combined score** |  | **Severity stage,**  **EPIC** | **PwD** | **Staff proxy** | **Combined score** |
| --- | --- | --- | --- | --- | --- | --- | --- | --- |
| All stages n=1086 | 0.77 (0.21) | 0.62 (0.24) | 0.68*  (0.22) |  | All stages n=760 | 0.80 (0.23) | 0.62 (0.36) | 0.61** (0.36) |
| CDR 1  n=770 | 0.78 (0.19) | 0.67 (0.21) | 0.75*  (0.20) |  | CDR 1  n=327 | 0.79 (0.24) | 0.71 (0.32) | 0.70** (0.31) |
| CDR 2  n=293 | 0.75 (0.24) | 0.50 (0.26) | 0.61*  (0.26) |  | CDR 2  n=323 | 0.81 (0.23) | 0.60 (0.35) | 0.61** (0.35) |
| CDR 3  n=16 | 0.66 (0.30) | 0.35 (0.27) | 0.49**  (0.20) |  | CDR 3  n=104 | 0.82 (0.20) | 0.38 (0.40) | 0.37**  (0.42) |
| ***Change in EQ-5D index score from T0 to T2 according to CDR stage*** | | | | | | | | |
| If CDR is stable  n=209 | -0.007 (0.19) | -0.043 (0.18) | -0.035 (0.18) |  | If CDR is stable  n=76 | -0.087 (0.28) | -0.045  (0.32) | -0.049 (0.32) |
| If CDR has progressed n=85 | -0.041 (0.20) | -0.131 (0.23) | -0.129 (0.21) |  | If CDR has progressed n=50 | -0.080 (0.24) | -0.179  (0.32) | -0.193 (0.29) |
| Values presented in this table use matched pair observations  *Combined score is statistically significantly different to PwD and proxy scores (p<0.05)  **Combined score is statistically significantly different to PwD score (p<0.05), but not proxy score (p>0.05) | | | | | | | | |

**Appendix 4**

Table A4) Final preferred EQ-5D-5L dimension models

| **EQ-5D dimension** | **ACTIFCARE** | **Model fit statistics** | **EPIC** | **Model fit statistics** |
| --- | --- | --- | --- | --- |
| **Mobility** | oprobit eq1SR_ i.eq1RP_ MMSE_Total_ PSMS_ PwD_age_ i.PwDs_sex, vce(cluster PatNo) | AIC: 1821.820; BIC: 1880.939  Ll(model): -898.910 | oprobit eq1staff_ i.eq1SR_ numeric_FAST_ age_ i.sex_, vce(cluster PatNo) | AIC: 2062.118; BIC: 2113.469  Ll(model): -1020.059 |
| **Self-care** | oprobit eq2RP_ i.eq2SR_ MMSE_Total_ PSMS_ PwD_age_ i.PwDs_sex_, vce(cluster PatNo) | AIC: 1820.145; BIC: 1879.216  Ll(model): -898.072 | oprobit eq2staff_ i.eq2SR_ numeric_FAST_ age_ i.sex_, vce(cluster PatNo) | AIC: 2260.686; BIC: 2312.023  Ll(model): -1119.343 |
| **Usual activities** | oprobit eq3RP_ i.eq3SR_ MMSE_Total_ NPI_ IADL_ PwD_age_ i.PwDs_sex_, vce(cluster PatNo) | AIC: 2594.979; BIC: 2658.41  Ll(model): -1248.489 | oprobit eq3staff_ i.eq3SR_ NPItotal_ numeric_FAST_ age_ i.sex_, vce(cluster PatNo) | AIC: 1443.861; BIC: 1499.633  Ll(model): -709.931 |
| **Pain/discomfort** | oprobit eq4SR_ i.eq5RP_ MMSE_Total_ PwD_age_ i.PwDs_sex_, vce(cluster PatNo) | AIC: 2070.851; BIC: 2124.978  Ll(model): -1024.425 | oprobit eq4SR_ i.merg_eq4staff i.sex_ age_, vce(cluster PatNo) | AIC: 1355.341; BIC: 1392.514  Ll(model): -668.671 |
| **Anxiety/depression** | oprobit eq5SR_ i.eq5RP MMSE_Total_ NPI_ PwD_age_ i.PwDs_sex_, vce(cluster PatNo) | AIC: 2022.598; BIC: 2081.646  Ll(model): -999.299 | oprobit eq5SR_ i.eq5staff_ NPItotal_ i.sex_ age_, vce(cluster PatNo) | AIC: 1123.015; BIC: 1174.351  Ll(model): -550.504 |

**Appendix 5**

Table A5a) Summary table of coefficients for the selected “mobility” models

| Regression variables | ACTIFCARE (target PwD) | EPIC (target staff proxy) |
| --- | --- | --- |
| Mobility (2) | 0.824* | 0.197* |
| Mobility (3) | 1.611* | 0.338* |
| Mobility (4) | 1.896* | 1.226* |
| Mobility (5) | 3.542* | 1.891* |
| Function | -0.123* | 0.358* |
| PwD age | 0.012* | 0.020* |
| PwD sex | 0.026 | 0.011 |
| MMSE | 0.045* | - |

Table A5b) Summary table of coefficients for the selected “self-care” models

| Regression variables | ACTIFCARE | EPIC |
| --- | --- | --- |
| Self-care (2) | 0.374* | -0.067 |
| Self-care (3) | 0.328* | 0.283* |
| Self-care (4) | 1.242* | 0.419* |
| Self-care (5) | 1.323* | 0.589* |
| Function | -0.599* | 0.603* |
| PwD age | -0.002 | 0.001 |
| PwD sex | 0.139 | 0.005 |
| MMSE | -0.006 | - |

Table A5c) Summary table of coefficients for the selected “usual activities” models

| Regression variables | ACTIFCARE | EPIC |
| --- | --- | --- |
| Usual activities (2) | 0.321* | 0.278* |
| Usual activities (3) | 0.542* | 0.500* |
| Usual activities (4) | 1.038* | 1.251* |
| Usual activities (5) | 0.980* | 1.141* |
| Function | -0.258* | 0.302* |
| NPI | 0.009 | 0.018* |
| PwD age | 0.002 | -0.004 |
| PwD sex | 0.262* | 0.128 |
| MMSE | -0.001 | - |

Table A5d) Summary table of coefficients for the selected “pain/discomfort” models

| Regression variables | ACTIFCARE (target PwD) | EPIC (target PwD) |
| --- | --- | --- |
| Pain/discomfort (2) | - | 0.320* |
| Pain/discomfort (3) | - | 0.795* |
| Pain/discomfort (4/5) | - | 1.122* |
|  | - |  |
| Anxiety/depression (2) | 0.596* | - |
| Anxiety/depression (3) | 1.022* | - |
| Anxiety/depression (4) | 1.415* | - |
| Anxiety/depression (5) | 2.073* | - |
| PwD age | 0.012* | -0.009 |
| PwD sex | 0.017 | -0.110 |
| MMSE | 0.020* | - |

Table A5e) Summary table of coefficients for the selected “anxiety/depression” models

| Regression variables | ACTIFCARE | EPIC |
| --- | --- | --- |
| Anxiety/depression (2) | 0.361* | 0.258* |
| Anxiety/depression (3) | 0.710* | 0.656* |
| Anxiety/depression (4) | 0.586* | 0.890* |
| Anxiety/depression (5) | 1.297* | 1.294 |
| NPI | -0.001 | 0.012* |
| PwD age | -0.001 | -0.004 |
| PwD sex | 0.008 | 0.156 |
| MMSE | -0.005 | - |

*p<0.05

**Appendix 6**

Figure A6a) “mobility” predictive margins for ACTIFCARE data


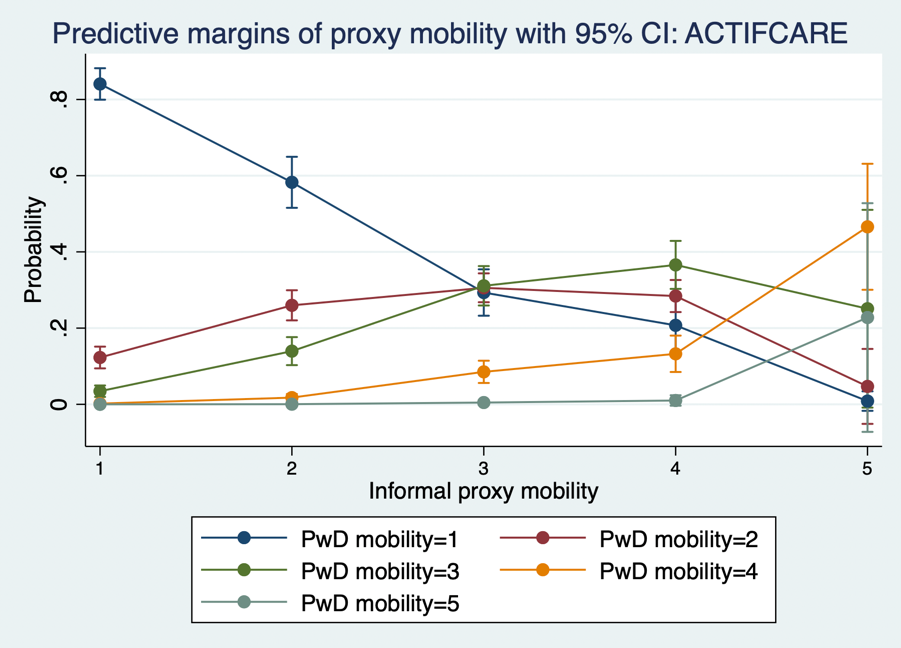


Figure A6b) “mobility” predictive margins for EPIC data


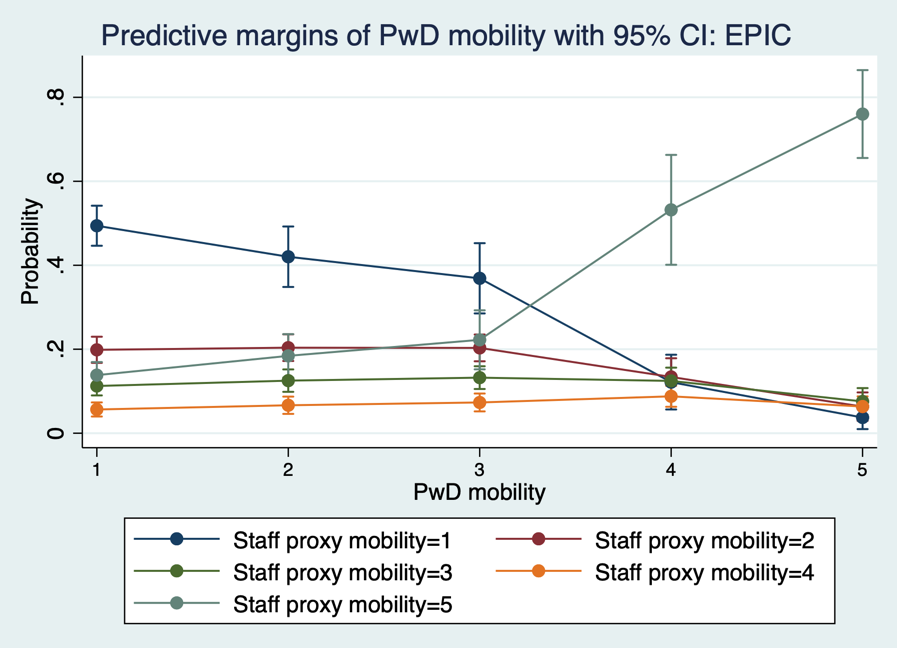


Figure A6c) “self-care” predictive margins for ACTIFCARE data


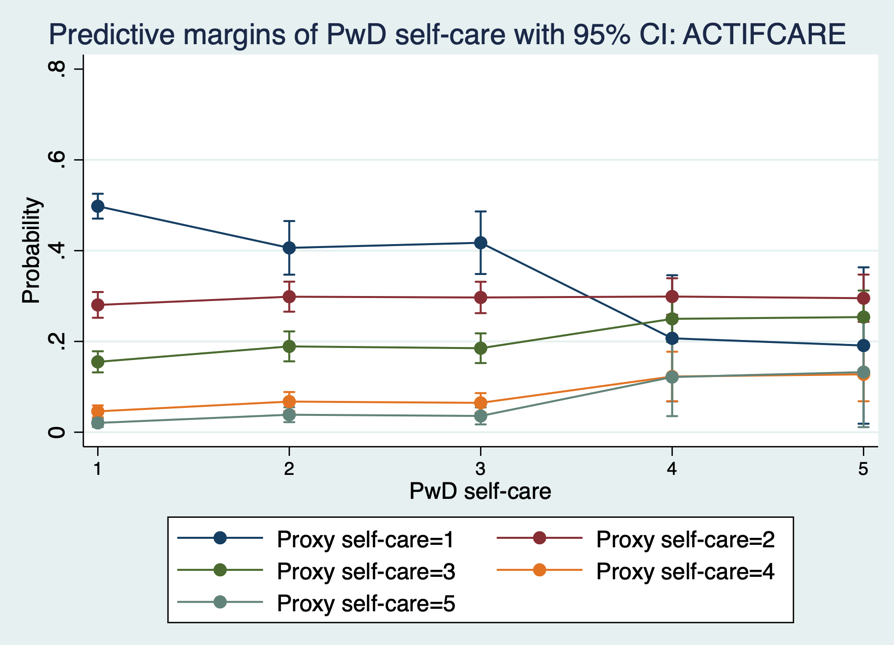


Figure A6d) “self-care” predictive margins for EPIC data

**
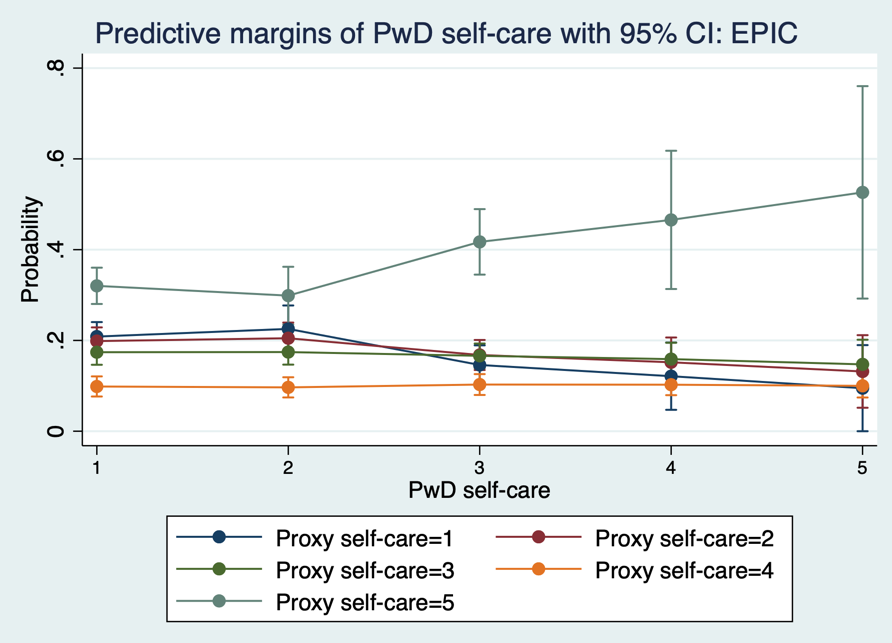
**

Figure A6e) “usual activities” predictive margins for ACTIFCARE data


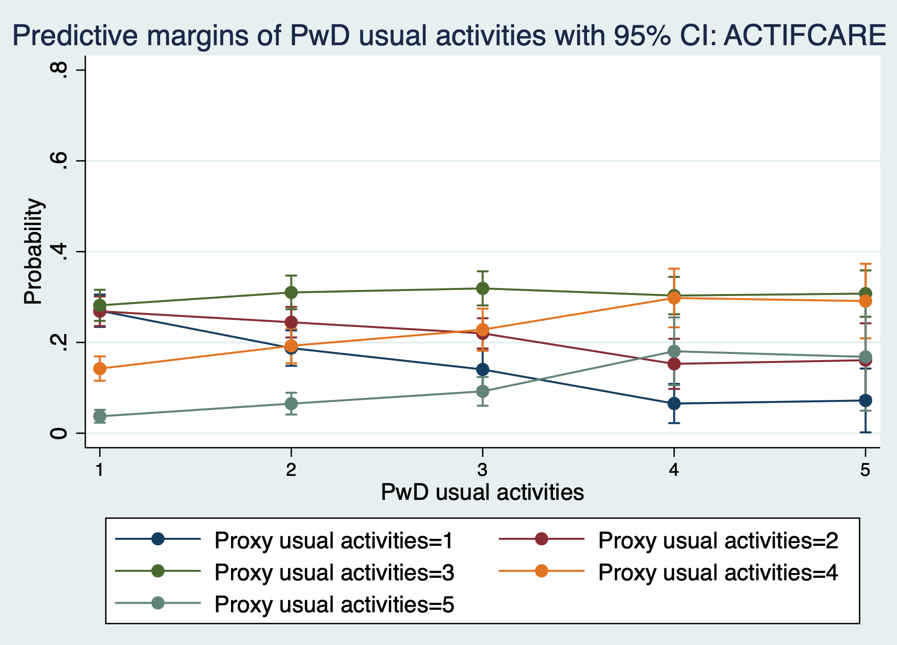


Figure A6f) “usual activities” predictive margins for EPIC data

**
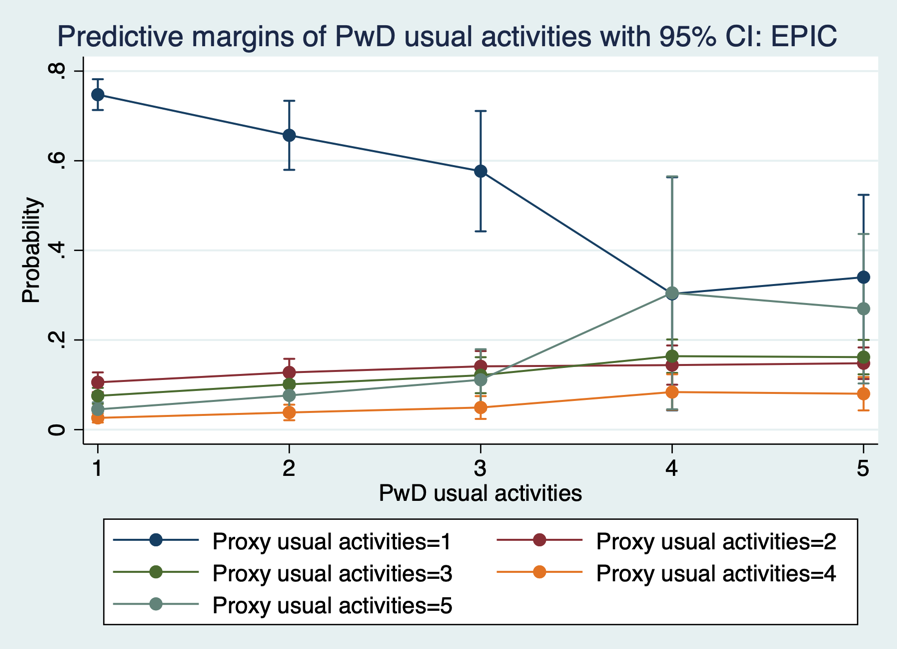
**

Figure A6g) “pain/discomfort” predictive margins for ACTIFCARE data

**
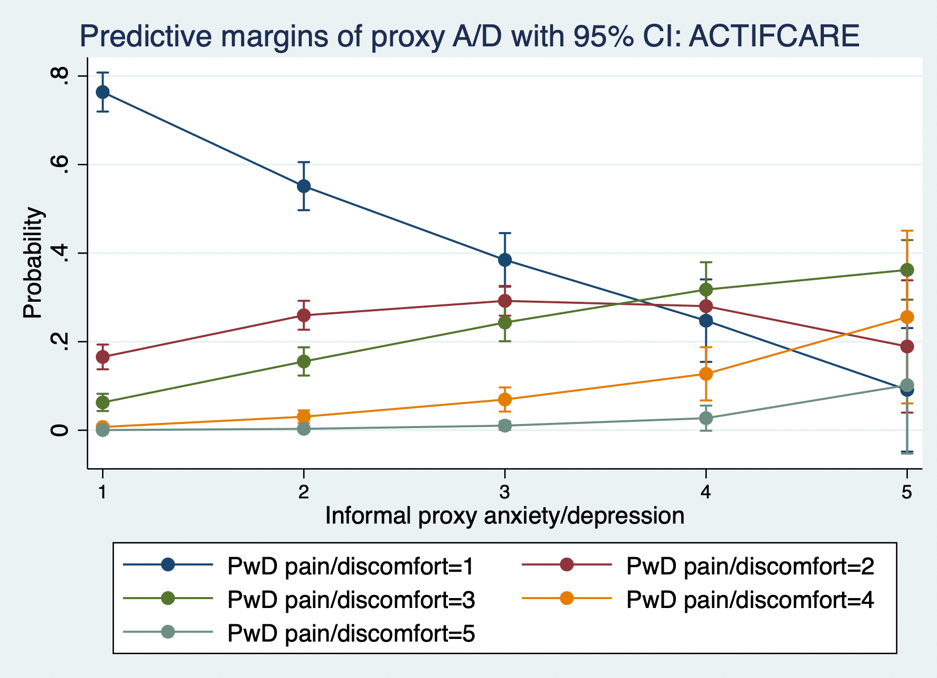
**

Figure A6h) “pain/discomfort” predictive margins for EPIC data

**
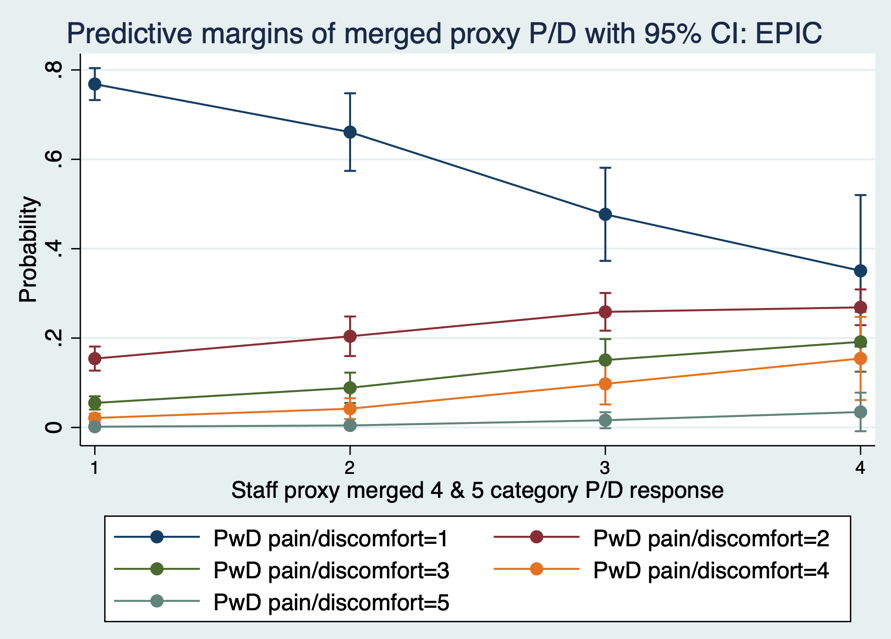
**

Figure A6i) “anxiety/depression” predictive margins for ACTIFCARE data


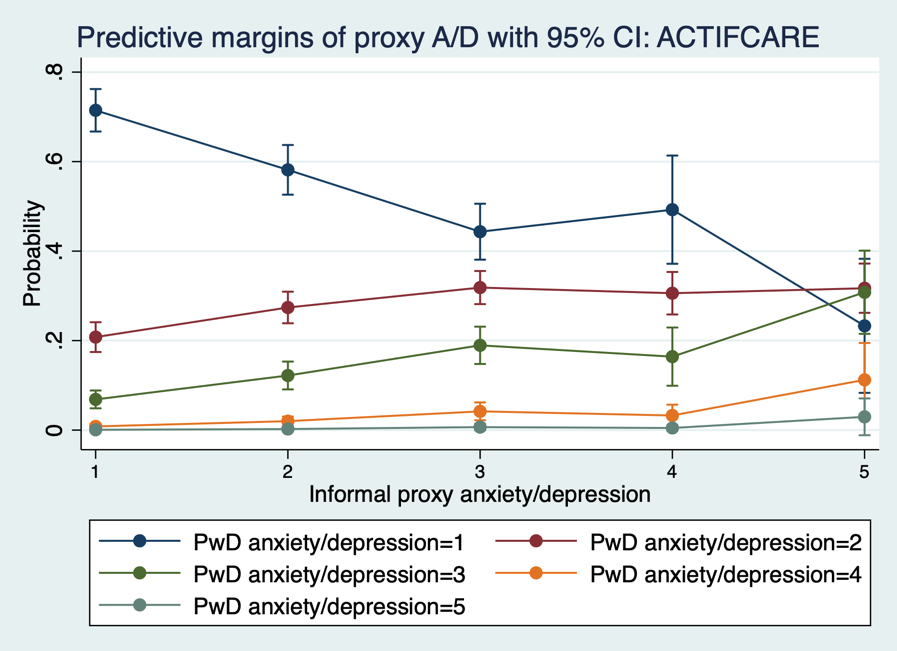


Figure A6j) “anxiety/depression” predictive margins for EPIC data


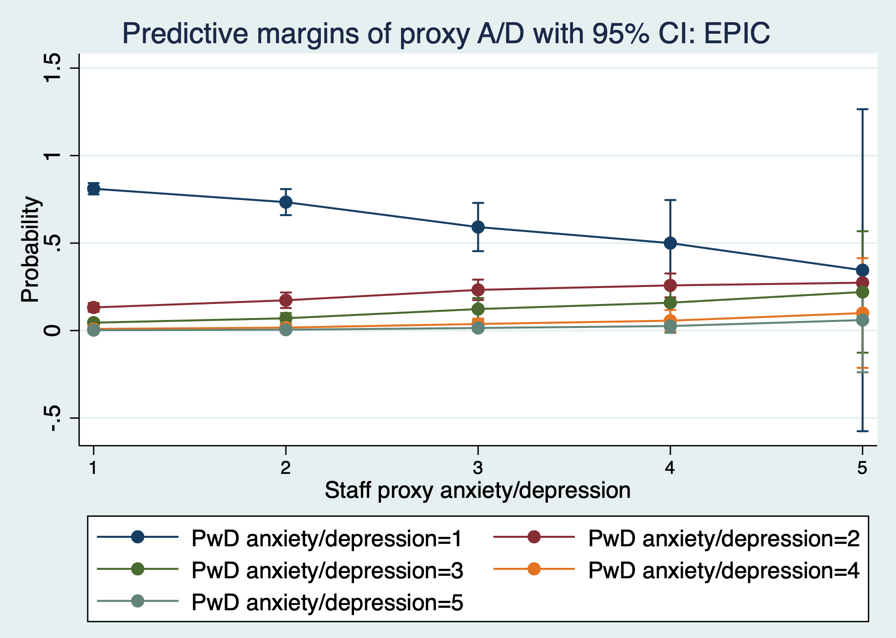


**Appendix 7**

Table A7) Summary of key findings

| **Key Findings** | **Implications** |
| --- | --- |
| Dementia severity influenced model performance, whereby optimality was seen for mild-to-moderate stages | Model predictions were less effective at extreme MMSE scores, influenced by low response proportions and highlighting challenges in severe dementia |
| Dimension-level differences in model performance were observed; strong alignment was seen for "anxiety/depression" and "pain/discomfort" predictions | Proxy EQ-5D data may more reliably predict PwD responses for specific dimensions |
| Choice of utility estimation method (2018 EQ-5D-5L vs. 5L to 3L value set) influences mean utility values | Researchers must carefully consider EQ-5D value set choice when estimating HRQoL using mapped “data” |
| Predicted utility distributions were narrower than observed data due to mapping analyses | Mapping models aim to estimate mean EQ-5D values, therefore minimising variability in scores |
| Community dwelling (ACTIFCARE) and residential care (EPIC) settings showed different model performances | Dimension-level mapping models may have increased relevance in community settings, but require further efforts to standardise data collection |
| The trial indicator variable showed significance in “self-care”, “usual activities” and “anxiety/depression” | Although it is not possible to determine the specific reason for the trial effects, it highlights the role that tailoring the dimension-models played |
| Both trials exhibit ceiling effects in reports, particularly for “pain/discomfort” and “anxiety/depression” | It is important to consider ceiling effects in interpretation of the findings, and to acknowledge that model performance is dependent on characteristics of the data |
| MMSE shows varied significance across dimensions | The presence of a cognitive measure in EPIC may have enhanced predictions for certain dimensions |

**Appendix 8**

Table A8) Number of utility observations per MMSE stage, ACTIFCARE

| MMSE stage | Utility Obs (n) |
| --- | --- |
| 0 | 2 |
| 1 | - |
| 2 | 1 |
| 3 | 3 |
| 4 | 1 |
| 5 | 4 |
| 6 | 4 |
| 7 | 8 |
| 8 | 15 |
| 9 | 16 |
| 10 | 21 |
| 11 | 26 |
| 12 | 39 |
| 13 | 36 |
| 14 | 54 |
| 15 | 49 |
| 16 | 52 |
| 17 | 58 |
| 18 | 56 |
| 19 | 69 |
| 20 | 72 |
| 21 | 79 |
| 22 | 66 |
| 23 | 53 |
| 24 | 52 |
| 25 | 29 |
| 26 | 38 |
| 27 | 26 |
| 28 | 19 |
| 29 | 7 |
| 30 | 7 |

**Appendix 9**

**EQ-5D-5L value set comparisons: ACTIFCARE**

**
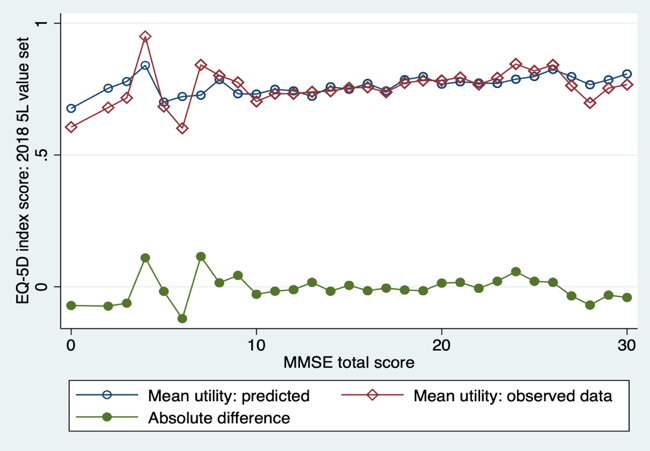

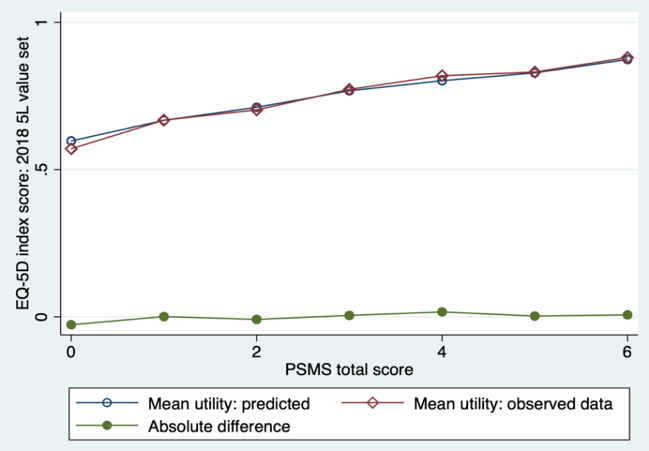
**

**
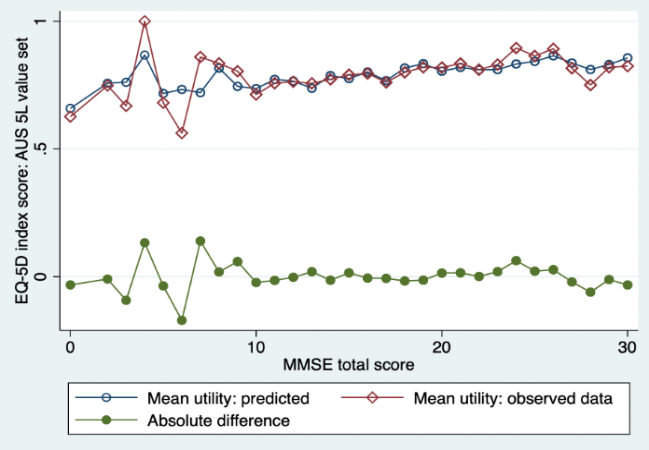

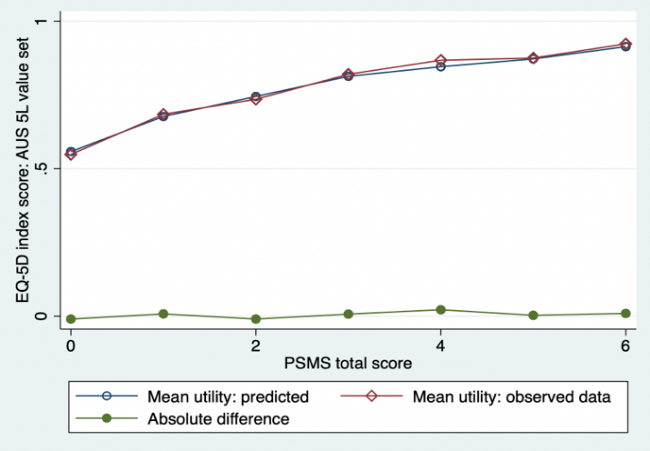
**

**
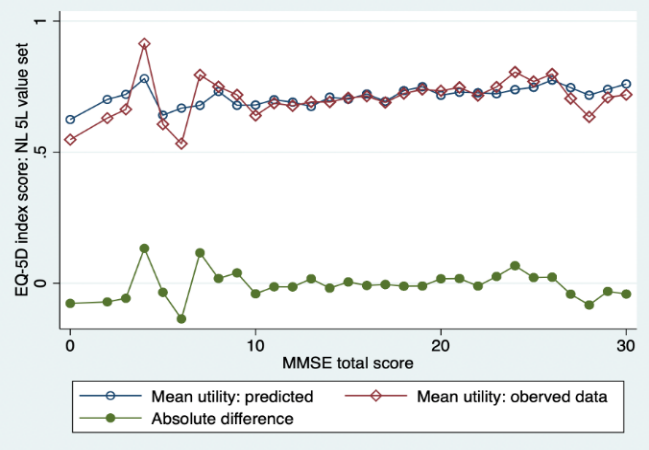

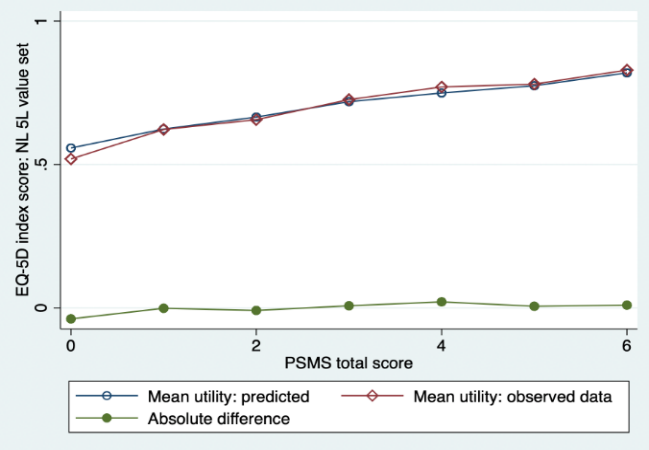
**

**
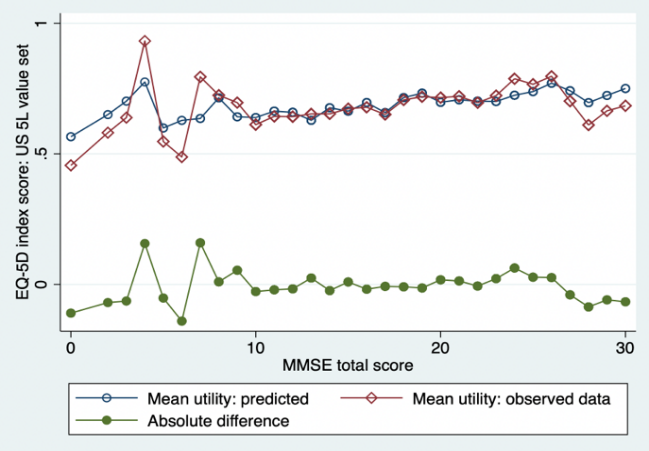

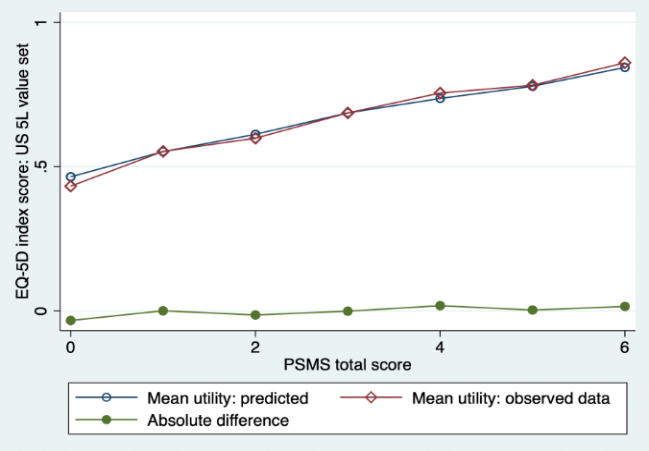
**

**EQ-5D-5L value set comparisons: EPIC**

**
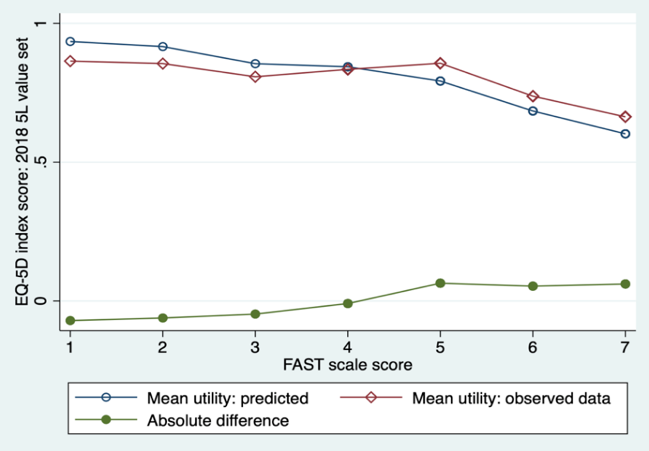
**


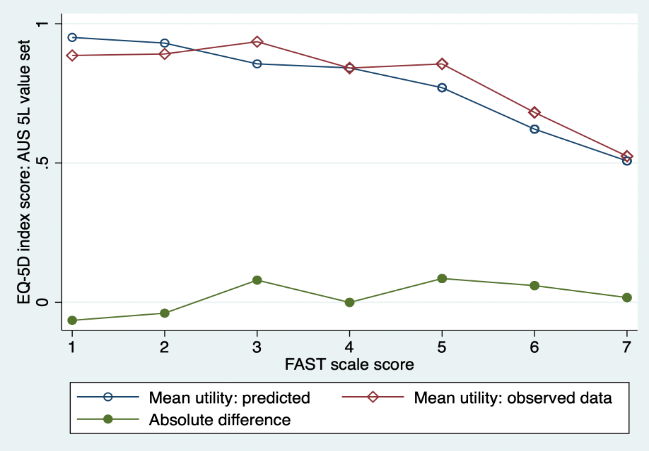


**
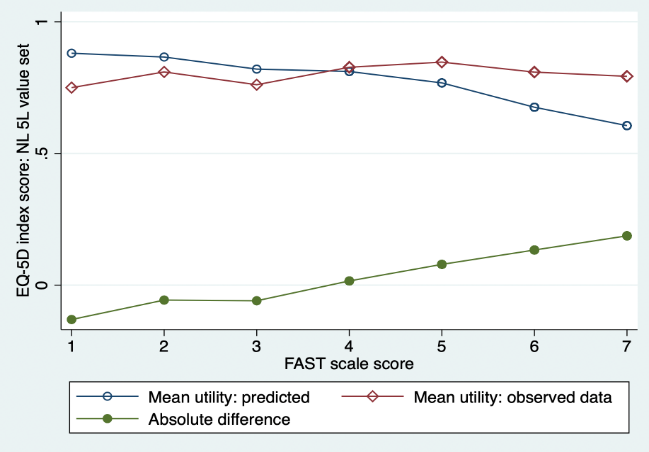
**


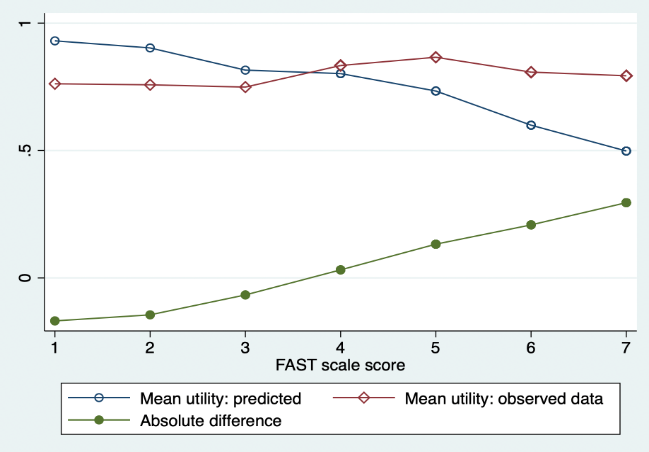

Supplement: Appendices [file mmc2.docx]
